# Effect of the Matrix Metalloproteinase Inhibitor Doxycycline on Human Trace Fear Memory

**DOI:** 10.1523/ENEURO.0243-22.2023

**Published:** 2023-02-23

**Authors:** Jelena M. Wehrli, Yanfang Xia, Benjamin Offenhammer, Birgit Kleim, Daniel Müller, Dominik R. Bach

**Affiliations:** 1 Department of Psychiatry, Psychotherapy and Psychosomatics, Psychiatric University Hospital Zurich, University of Zurich, Zurich 8032, Switzerland; 2 Experimental Psychopathology and Psychotherapy, Department of Psychology, University of Zurich, Zurich 8050, Switzerland; 3 Department of Clinical Chemistry, University Hospital Zurich, University of Zurich, Zurich 8091, Switzerland; 4 Wellcome Centre for Human Neuroimaging and Max Planck UCL Centre for Computational Psychiatry and Ageing Research, University College London, London WC1B 5EH, United Kingdom; 5 Hertz Chair for Artificial Intelligence and Neuroscience, Transdisciplinary Research Area “Life and Health,” University of Bonn, Bonn 53121, Germany

**Keywords:** doxycycline, fear conditioning, memory modification, MMP-9, trace fear memory

## Abstract

Learning to predict threat is of adaptive importance, but aversive memory can also become disadvantageous and burdensome in clinical conditions such as posttraumatic stress disorder (PTSD). Pavlovian fear conditioning is a laboratory model of aversive memory and thought to rely on structural synaptic reconfiguration involving matrix metalloproteinase (MMP)9 signaling. It has recently been suggested that the MMP9-inhibiting antibiotic doxycycline, applied before acquisition training in humans, reduces fear memory retention after one week. This previous study used cued delay fear conditioning, in which predictors and outcomes overlap in time. However, temporal separation of predictors and outcomes is common in clinical conditions. Learning the association of temporally separated events requires a partly different neural circuitry, for which the role of MMP9 signaling is not yet known. Here, we investigate the impact of doxycycline on long-interval (15 s) trace fear conditioning in a randomized controlled trial with 101 (50 females) human participants. We find no impact of the drug in our preregistered analyses. Exploratory *post hoc* analyses of memory retention suggested a serum level-dependent effect of doxycycline on trace fear memory retention. However, effect size to distinguish CS+/CS− in the placebo group turned out to be smaller than in previously used delay fear conditioning protocols, which limits the power of statistical tests. Our results suggest that doxycycline effect on trace fear conditioning in healthy individuals is smaller and less robust than anticipated, potentially limiting its clinical application potential.

## Significance Statement

The inhibition of matrix metalloproteinase (MMP)9 attenuates memory consolidation and subsequent recall in a delay cue conditioning paradigm. However, it is currently unclear whether this is also the case for other learning scenarios that rely on a different neurocircuitry. We test this hypothesis in human trace fear conditioning which employs remote cues and is additionally dependent on hippocampus involvement. We find that doxycycline does not reduce fear retention in our preregistered analyses. Exploratory analyze might potentially suggest a weak effect of doxycycline on trace fear memory retention.

## Introduction

The ability to predict threat is fundamental for survival and requires remembering predictive cues. However, when threat is absent, lingering aversive memory can contribute to trauma-related clinical conditions (Iyadurai et al., 2019). Even the most successful treatments for these conditions, which focus on trauma memory modulation (Watkins et al., 2018), leave room for improvement (Yehuda et al., 2015). The development and refinement of interventions for treating maladaptive trauma memory in the laboratory is often based on Pavlovian fear conditioning (Pape and Pare, 2010), also termed threat conditioning (LeDoux, 2014). One goal is to prevent or attenuate experimentally induced fear memory (Kroes et al., 2015), while it is labile and not yet consolidated. So far, pharmacological options for attenuating fear memory in humans are limited. Oral administration of the β-blocker propranolol shortly before memory consolidation has been tested in clinical studies with some success (Grillon et al., 2004; Elsey et al., 2020). Intrahippocampal infusion of GABA agonists (such as benzodiazepines) in nonhumans (Gafford et al., 2005), or oral benzodiazepines in humans, can disrupt fear conditioning (Brignell and Curran, 2006). Evidence for clinical effectiveness of benzodiazepines in posttraumatic stress disorder (PTSD) prevention is mixed (Guina et al., 2015; Campos et al., 2022).

The molecular processes supporting fear memory acquisition and consolidation in the amygdala (Schafe and LeDoux, 2000) are assumed to be similar to those generated by long-term potentiation (LTP; LeDoux, 2000). *In vitro* studies have revealed two distinct temporal phases, which rely on different molecular mechanisms. First, early phase LTP (E-LTP), lasting only minutes to hours, and secondly, late phase LTP (L-LTP) which lasts hours to days, and involves structural reconfiguration of the synapse (Frey et al., 1993). The precise molecular signaling pathways eliciting synaptic reconfiguration are not fully known, but have been shown to involve matrix metalloproteinase (MMP)9 (Huntley, 2012; Beroun et al., 2019). Blocking MMP9 reduces L-LTP (Nagy, 2006; Wang et al., 2008; Gorkiewicz et al., 2015) and can reduce the behavioral expression of learning (S.E. Meighan et al., 2006; Wright et al., 2007). By blocking MMP9 during memory consolidation of a traumatic experience, the development of trauma-related disorders could potentially be prevented. Specific MMP9 inhibitors are not currently approved for use in humans, but the antibiotic doxycycline is an inhibitor of several MMPs, including MMP9 (Golub et al., 1991; Hanemaaijer et al., 1998; Kim et al., 2005). Doxycycline crosses the blood-brain barrier (Mento et al., 1969) and has recently been shown to reduce retention of fear memory after one week, when applied before fear acquisition training (Bach et al., 2018b).

This previous study used cued delay fear conditioning as an experimental model, in which threat predictor and aversive outcome are simultaneously presented. Yet, intrusive memory and physiological arousal after psychological trauma cannot only be triggered by stimuli present during trauma, but also by those that occurred at some interval before the traumatic event (Ehlers et al., 2002). In Pavlovian conditioning terminology, individuals with PTSD experience intrusions both of the conditioned stimulus (CS), i.e., cues and contexts accompanying the trauma, and of the unconditioned stimulus (US), i.e., the traumatic event itself (Hackmann et al., 2004; Franke et al., 2021).

In the laboratory, prediction by temporally preceding events is modelled in trace fear conditioning, where CS and US are separated in time (Rescorla, 1988; Sehlmeyer et al., 2009; Mertens et al., 2020). Crucially, there are substantial differences in the neural circuits that support these two types of learning. Delay fear conditioning is known to require synaptic plasticity in lateral and central amygdala (Ciocchi et al., 2010), and can be acquired in the absence of a functional hippocampus (Solomon et al., 1986). Trace fear conditioning requires hippocampal neurons (Gilmartin et al., 2012). This is likely the case for associating preceding cues with psychological trauma as well. Hence, it appears important to further test candidate procedures for memory modification in trace fear conditioning protocols, as it is unclear how hippocampus-dependent consolidation would be affected by the MMP9 inhibitor doxycycline. Although inhibition of MMP9 of specific areas of the cornu ammonis (CA) appeared to reduce L-LTP in animal studies (P.C. Meighan et al., 2007; Wójtowicz and Mozrzymas, 2010), the behavioral effects of such intervention in humans remains elusive.

## Materials and Methods

### Overview

We tested the impact of doxycycline versus placebo on human trace fear conditioning in a randomized, placebo-controlled, double-blind trial. We used a trace interval of 15 s, which is long enough to require hippocampus involvement in rodents (Chowdhury et al., 2005). Memory retention was tested one week later, after drug wash-out. Our primary memory measure during the recall test was based on fear-potentiated startle eye-blink response (SEBR), which our previous work had identified as the most sensitive index of fear memory retention in general (Khemka et al., 2017) and in the presently used paradigm in particular (Wehrli et al., 2022). As secondary outcome, we recorded skin conductance responses (SCRs) during the trace interval. As the recall test did not involve any US, extinction might have occured, which was taken into account for the analysis. While the presentation of startle probes may alter the extinction process, there is no evidence that it inhibits extinction (Sjouwerman et al., 2016). Because startle probes can impair learning (Sjouwerman et al., 2016), they were not included during acquisition training. Instead, acquisition was quantified using SCR and pupil dilation [pupil size responses (PSRs)].

### Participants

We recruited 101 participants from the general population between November 5, 2019 and December 22, 2020 and randomly assigned them to placebo (*n* = 50, 25 females) or doxycycline (*n* = 51, 25 females). Three participants did not take part in recall visit 3 as they were obliged to self-isolate because of the COVID-19 pandemic. One further participant did not complete visit 2 per protocol (no US delivery because of equipment failure). The reported final sample therefore includes 97 participants, *n* = 48 in the placebo group (24 females) and *n* = 49 in the doxycycline group (25 females). There were no differences between groups in age, sex, body mass index (BMI), baseline personality measures and US intensity (see Table 1).

**Table 1:** Sample characteristics

	Placebo		Doxycycline			
Sex	Male (*n* = 24)	Female (*n* = 24)	Male (*n* = 24)	Female (*n* = 25)		
	Mean	SD	Mean	SD	*p*-value	Cohen’s *d*
Age (years)	24.98	4.04	24.35	3.76	0.43	0.16
Weight (kg)	71.86	11.74	69.44	13.55	0.35	0.19
US intensity (mA)	4.88	2.97	5.13	2.74	0.67	0.09
Pain ratings pre vs post	13.06	13.43	8.10	14.21	0.080	0.36
Accuracy acquisition (%)	96.93	3.54	97.35	3.97	0.58	0.11
Accuracy recall (%)	96.18	5.54	97.35	4.36	0.25	0.23
Response rate acquisition (%)	98.65	2.52	98.83	2.80	0.74	0.07
Response rate recall (%)	98.82	2.00	99.12	2.02	0.47	0.15
Differential arousal acquisition (%)	43.09	36.80	31.75	41.06	0.16	0.29
Differential arousal recall (%)	17.59	31.03	13.71	27.74	0.52	0.13
Differential valence acquisition (%)	−40.68	36.06	−37.60	41.53	0.70	0.08
Differential valence recall (%)	−18.30	29.30	−12.93	26.05	0.34	0.19
State anxiety preacquisition	31.65	6.37	30.53	5.46	0.37	0.19
State anxiety prerecall	31.65	6.72	28.09	4.38	0.004*	0.62
Trait anxiety preacquisition	30.94	6.53	30.29	6.88	0.64	0.10
BDI screening	3.02	3.09	2.73	2.21	0.60	0.11
BDI postrecall	2.92	4.21	3.02	3.63	0.90	0.03

US intensity: electric current used in experiment; pain ratings pre vs post: difference in average pain ratings of 14 stimuli before and after the acquisition test; accuracy: % of correct responses in identification task, average of acquisition (visit 2) and recall (visit 3); performance: % of responses in identification task, average of acquisition (visit 2) and recall (visit 3); arousal: difference in arousal ratings between CS+/CS− after the acquisition session (visit 2), and after the recall session (visit 3); valence: difference in valence ratings between CS+/CS− after the acquisition session (visit 2), and after the recall session (visit 3); state anxiety: measured with State-Trait Anxiety Inventory (STAI; Laux et al., 1981); trait anxiety: measured with State-Trait Anxiety Inventory (STAI; Laux et al., 1981); BDI: Beck Depression Inventory (Beck and Hautzinger, 2001); *p*-value: two-sample *t* test; Cohen’s *d*: Cohen’s *d* effect size of the group difference. SD: standard deviation; ‘*’ significant (*p* < 0.05) difference between placebo and doxycycline group.

The study was conducted in accordance with the Declaration of Helsinki and approved by the governmental research ethics committee (Kantonale Ethikkomission Zürich KEK-ZH-2018–01973) and the Swiss Agency for Therapeutic Products (Swissmedic; 2019DR1026). All participants gave written informed consent before the experiment using a form approved by the ethics committee. The study was preregistered with a WHO-approved primary registry (German Clinical Trials Register, DRKS00017037) and at the Swiss Federal Complementary Database (Kofam: SNCTP000003485). During recruitment, the analysis protocol was adapted based on ongoing methodological work (Wehrli et al., 2022). The final analysis protocol was preregistered on OSF (https://osf.io/uqtr5/) on December 20, 2020 before unblinding the study medication.

### Power analysis

To determine required sample size, we conducted a power analysis (using G*power) based on a methodological study in which the effect size for differential SEBR in an untreated control group was (Cohen’s) *d* = 1.17 (Khemka et al., 2017). Assuming equal variance in the doxycycline-treated group and a best-case scenario of no variation in the treatment effect (for details, see Bach et al., 2020), a 50% fear memory reduction would correspond to an effect size of *d* = 0.59. Thus, a minimum sample size of *N* = 74 was required to achieve 80% power at an α level of 0.05. We recruited *N* = 101 participants to compensate for (unknown) treatment variance and potential dropouts.

### Study medication

The study medication was the tetracycline antibiotic doxycycline, brand name Vibramycin (Pfizer). Study dose (200 mg) was based on a previous study using delay fear conditioning (Bach et al., 2019). Doxycycline demonstrably penetrates the blood-brain barrier (Mento et al., 1969) and is clinically used to treat neuroborreliosis (Dotevall and Hagberg, 1989). During treatment of borreliosis, doxycycline is detectable in cerebrospinal fluid, both 4 h after ingestion on treatment day 13 (200 mg, orally every 24 h; Karlsson et al., 1996) and 2–3 h after the last administration on treatment days 5–8 (100 or 200 mg, orally twice a day; Dotevall and Hagberg, 1989). For consistency with previous studies (Bach et al., 2018b, 2019), we scheduled fear memory acquisition ∼3.5 h after drug ingestion.

According to the manufacturer’s information, the drug’s half-life is ∼16 h. Hence, the drug was cleared >99.9% before the recall session 7 d after ingestion. The drug was manufactured, blinded, and randomized separately for males and females, by a GMP-licensed pharmacy (Kantonsapotheke Zurich). Mannitol was used as placebo. Randomization was broken after the last participant completed the study, data were checked for consistency, and the study analysis plan was preregistered on OSF.

### Experimental design

#### Screening visit 1 (day −14 to day −2)

**Figure 1.**
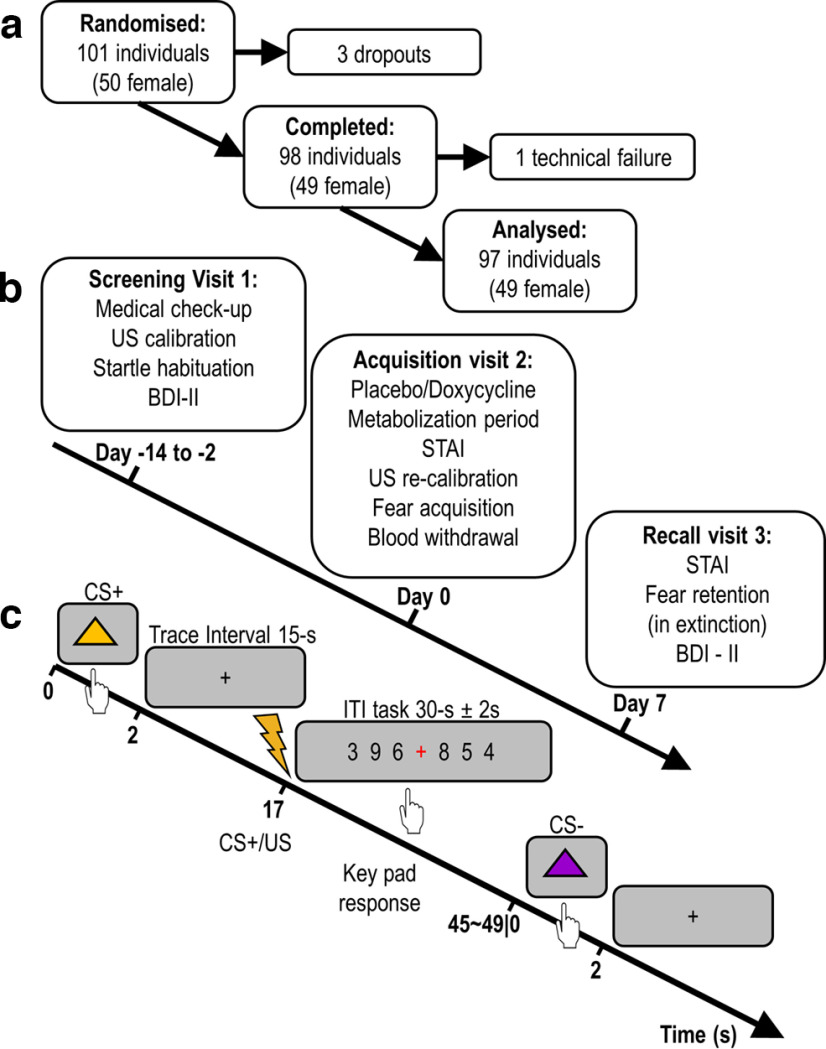
Experimental protocol. ***a***, Recruitment and exclusion of participants. ***b***, Study visit timeline. ***c***, Intratrial procedure. A CS (triangles) was presented for 2 s, participants responded with a key press to indicate which CS color was presented; 100% of CS+ were followed by a 1-s US (painful electric stimulation), each ITI trial involved a simple attention task, presenting single digits with a red cross in between, participants were asked to respond to the presentation of the red cross with a key press. CS, conditioned stimulus; US, unconditioned stimulus.

The study procedure is illustrated in Figure 1*b*. On screening visit 1, participants were medically screened by the study physician to check exclusion criteria, and weight/height was measured to compute BMI. Participants were screened for depression using Beck’s Depression Inventory (Beck and Hautzinger, 2001), using a cutoff of 14 points which would indicate mild depressive symptoms. Additionally, individual US intensity was calibrated, and habituation startle sounds were presented.

#### Acquisition visit 2 (day 0)

Acquisition visit 2 started in the morning hours between 7:30 to 11:00 A.M. Before ingestion of the study medication, participants were asked about their health status, medication intake and psychotropic substance consumption since the screening visit. Then they were administered the study medication. Participants were asked not to eat, or drink beverages containing milk, in the hour before and after drug ingestion, as this can influence the absorption of doxycycline (Meyer et al., 1989). During a 180-min metabolization interval, participants were monitored by study staff. Following this, participants filled in the German translation of the State-Trait Anxiety Inventory (STAI; Laux et al., 1981). Then US intensity was recalibrated. Approximately 210 min after drug intake, the fear acquisition protocol started, lasting ∼40 min. Afterwards, participants were asked to indicate CS-US contingency for each CS from 0% to 100% (0 = never received a shock, 100 = always received a shock), as well as their arousal (0–100%, 0 = very calm, 100 = very excited) and valence (0–100%, 0 = very negative, 100 = very positive) for each CS. This was followed by a neuropsychological test in the context of a different study, which will be reported elsewhere. At the end of the session, ca. 360 min after drug-intake, venous blood samples were taken (16 ml) to establish doxycycline serum level.

#### Recall visit 3 (day +7)

In recall visit 3, participants filled in the state part of the STAI (Laux et al., 1981), then US electrodes were attached, and the participants were seated in the same experimental room as in visit 2 for the fear recall test. Afterwards, they indicated the CS-US contingency during the recall test as well as their valence and arousal for each CS, and then their memory of the CS-US contingency during the acquisition session.

### Task and stimuli

Fear acquisition training comprised 40 trials (20 CS+, 20 CS−), and the fear recall test 30 trials (15 CS+, 15 CS−). Each CS was followed by a 15-s trace interval. During acquisition training, US was presented after the trace interval (15 s after CS offset) in all CS+ trials (100% reinforcement; Fig. 1*c*). During recall test no US was presented, and a white noise startle probe was delivered on each trial 13 s after CS offset (i.e., 2 s before the expected US delivery), both in CS+ and CS− trials. Before the recall test, participants were instructed that they might receive a US. Trials were separated by a 30-s intertrial interval (ITI), with a ±2-s jitter, during which they were given an incidental task (see below).

CS were two differently colored [yellow (RGB: 225, 224, 177) and purple (RGB: 238, 194, 244)] isoluminant triangles, presented for 2 s at the center of an isoluminant gray (RGB: 175, 175, 175) computer screen at a visual angle of ∼4.1°. Association of CS+/CS− to CS color was counterbalanced across participants. As an identification task, participants were asked to indicate the color of the CS by pressing the left/right cursor keys during CS presentation on a standard computer keyboard. If participants gave the wrong or no response, the words “wrong key” and “no response,” respectively, were presented immediately after CS offset. During the 15-s trace interval, a white (RGB: 255, 255, 255) fixation cross was presented at the center of the gray background screen at a visual angle of ∼0.8°.

US consisted of a sequence of 83 square electric pulses of 0.2-ms duration with a duty cycle of 1.67%, summing up to a total duration of 1000-ms. US were delivered to the participants’ dominant forearm via a pin-cathode/ring anode configuration. Electric pulses were generated by a constant current stimulator (Digitimer DS7A, Digitimer). Intensity of the US was set to a perceived intensity between 80–90% of the lowest clearly painful stimulus. US intensity was estimated in three phases. In the first phase, US intensity was increased until a painful level was firmly reached, marking the upper limit for the second phase, during which 14 US with random intensities were delivered. Participants were asked to rate their subjective pain perception for each of them from 0 to 100. These ratings were then linearly interpolated to estimate a US intensity corresponding to 90% of a clearly painful stimulus. Stimuli with this intensity were once more presented to the participants and adjusted if necessary.

During the ITI, a simple visual detection task was presented to keep participants attentive, because previous studies showed that participants become drowsy even with shorter ITIs (15 s; de Haan et al., 2018). Thirteen white (RGB: 255, 255, 255) single-digit numbers were presented at a rate of 1 Hz for 0.2 s each. Embedded in the stream of white numbers, a red (RGB: 255, 0, 0) fixation cross was presented to which participants were asked to respond via key press. The onset of the task was randomized between 5 and 10 s after US offset. Congruent tasks during fear conditioning (both in delay and trace conditioning) might reduce fear learning, however the extent of the reduction is dependent on the cognitive load involved (Carter et al., 2003). To reduce interference with fear learning we employed a simple visual detection task which requires minimal attention and working memory.

In the recall session, white noise startle probes of 20 ms duration, instantaneous rise time, and 102-dB loudness, were delivered binaurally via headphones (HD 202, Sennheiser). The experiment was conducted in a dark, soundproof chamber. The experimental task was presented on a Dell P2014h 20-inch screen, set to an aspect ratio of 4:3 at 60 Hz, with a resolution of 1152 × 864 pixels. Participants’ heads were positioned with a chin rest at 70 cm distance from the monitor and 47 cm from the eye tracker.

### Psychophysiological recordings

Electromyogram (EMG) was recorded from the orbicularis oculi muscle of the participants’ left eye, with two 4 mm Ag/AgCl cup electrodes filled with high-conductance gel. The electrodes were positioned below the lower eyelid on the muscle, in a vertical line with the pupil in forward gaze, and below the lateral canthus. Electromyogram was amplified with a gain of 2000, low-pass filtered at 1 Hz and high-pass filtered at 500 Hz (EMG100C, Biopac Systems). Skin conductance was recorded with a 0.5-V constant voltage (EDA100C, Biopac Systems) from the thenar/hypothenar of the nondominant hand, with disposable Ag/AgCl snap electrodes (EL507, Biopac Systems), filled with 0.5% NaCl electrolyte gel (Hygge and Hugdahl, 1985; GEL101, Biopac Systems). A ground electrode was placed on the nondominant elbow. We recorded electrocardiogram (ECG) with pregelled disposable Ag/AgCl snap electrodes (01-7500, TIGA-MED), which were placed on both wrists and above the right ankle. Lead I configuration was generated and amplified (ECG100C, Biopac Systems). To track respiration, a single-belt cushion system (RSP100C, Biopac Systems) was used. All signals were digitized at 2000 Hz (MP160, Biopac Systems) and recorded (Acknowledge, Biopac Systems). We recorded pupil diameter and gaze direction with an EyeLink 1000 System (SR Research) at a sampling rate of 500 Hz. A nine-point protocol implemented in the EyeLink 1000 software was used to calibrate gaze direction.

### Preparation and storage of blood samples

Within an hour of withdrawal, two serum tubes (8 ml each) of blood samples were centrifuged in a Universal 320 R (Hettich) for 10 min at 2800 × *g* and 4°C. After centrifugation, 2 × 2 ml serum was pipetted and stored at −80°C. After unblinding the randomization, samples of participants in the doxycycline group were analyzed. One blood sample from the doxycycline group was missing, because of fainting of the participant during blood withdrawal. Doxycycline was measured using liquid chromatography coupled to high-resolution mass spectrometry (LC-HRMS) on a Q Exactive system (Thermo Fisher Scientific). After addition of the internal standard demelocycline, protein precipitation and centrifugation, samples were directly injected. As stationary phase, a Hypersil Gold C8 column (100 × 3 mm) was used, mobile phases consisted of 10 mmol/l ammoniumacetate in methanol/acetonitrile (50/50 v/v) plus 0.1% formic acid and 10 mmol/l aqueous ammonium acetate plus 0.1% formic acid.

### Data analysis

#### Overview

For the recall test, an updated data analysis plan was preregistered on OSF (https://osf.io/uqtr5/) before the last person completed the study and before unblinding the drug randomization. This was based on methodological work with the same experimental paradigm, which had identified SEBR as the only index with sensitivity to detect trace fear memory retention on day +7 (Wehrli et al., 2022). This work also identified PSR and SCR as indices of trace fear acquisition. Here, we used the same preprocessing and scoring methods as in this previous work. We defined SEBR as primary outcome measure, and SCR, pupil dilation, fear-conditioned bradycardia, and respiration amplitude, as secondary outcome measures. A priori, we did not expect cardiac and respiratory conditioned responses, based on our previous methodological work (Wehrli et al., 2022). An exploratory analysis in the placebo group confirmed the lack of conditioned responses (Extended Data Figs. 3-1 and 3-2), and consequently these measures were not analyzed further.

10.1523/ENEURO.0243-22.2023.f3-1 Extended Data Figure 3-1 Acquisition paired t test CS+/CS−, not corrected for multiple comparisons. Download Figure 3-1, DOC file.

**Figure 2.**
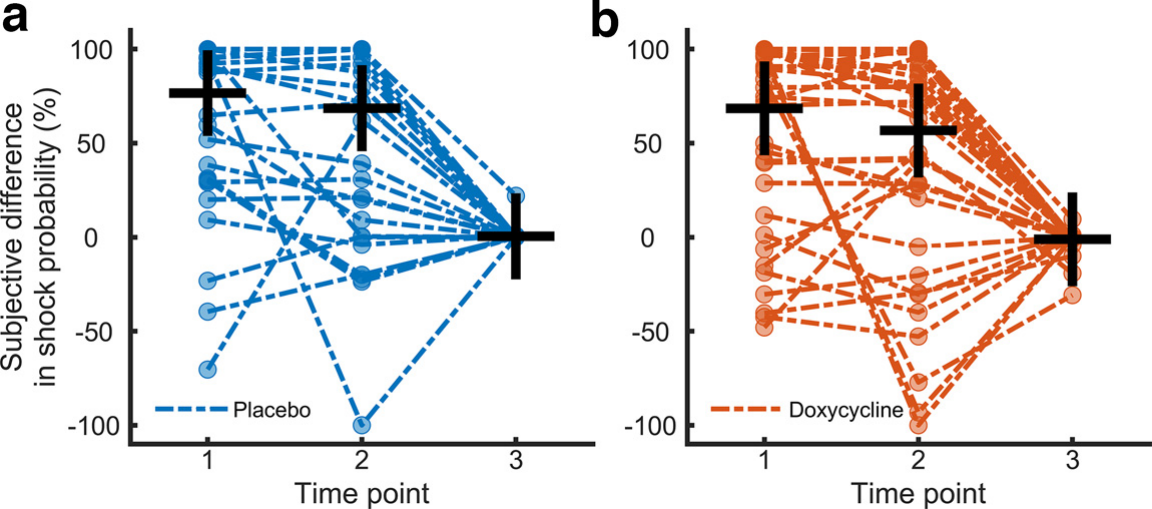
Contingency ratings, displayed as CS+/CS− differences. CS+/CS− difference in objective shock probability is 100% in acquisition and 0% in recall session. ***a***, Placebo group, individual ratings depicted in blue. ***b***, Doxycycline group. Individual ratings depicted in red. Mean values are marked with a horizontal black line, standard deviation is depicted with a vertical line. Time points: 1, directly after the acquisition phase; 2, acquisition contingency as remembered after the recall session; 3, after the recall session.

**Figure 3.**
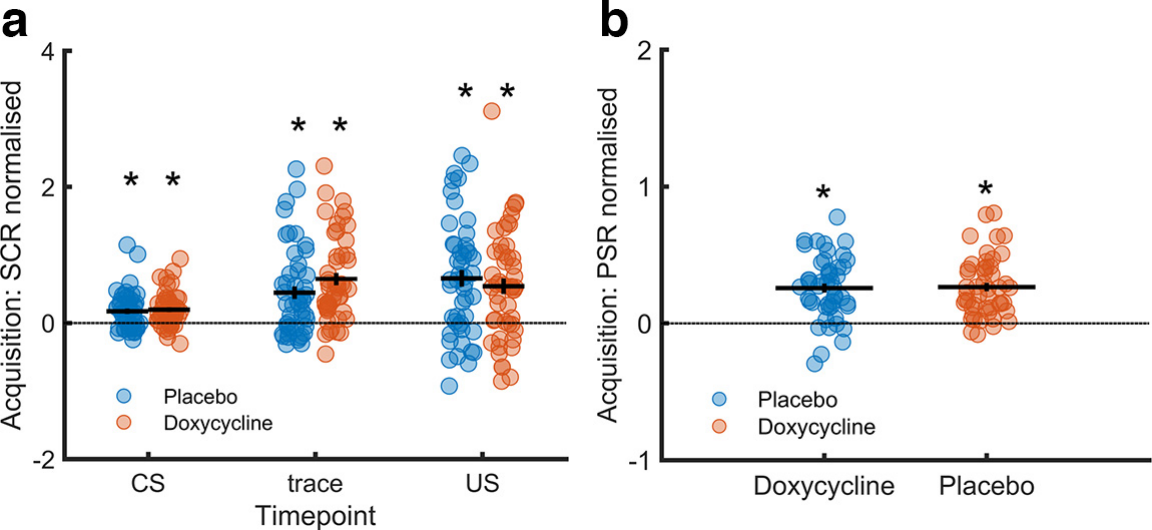
CS+/CS− differences in skin conductance responses (SCR) and pupil size responses (PSR) during acquisition training. ***a***, Normalized SCR difference between CS+/CS− in the acquisition session, averaged over all trials. CS: to CS presentation, trace: during trace interval, US: to US presentation. ***b***, Normalized PSR difference between CS+/CS− in the acquisition session, averaged over all trials. Average values are depicted with a horizontal line, standard error of mean (SEM) with a vertical line, scatterplot shows individual values, asterisk denote significant (*p* < 0.05) difference between CS+ and CS− trials. Detailed comparisons in Extended Data Figures 3-1 and 3-2. For sex-specific differences, see Extended Data Figures 3-3 and 3-4.

10.1523/ENEURO.0243-22.2023.f3-2 Extended Data Figure 3-2 Acquisition independent t test between CS+/CS− difference for placebo and doxycycline group, not corrected for multiple comparisons. P = Placebo, D = Doxycycline Download Figure 3-2, DOC file.

10.1523/ENEURO.0243-22.2023.t3-1 Extended Data Table 3-1 SCR to CS LME (estimated with “nlme” package) in fear acquisition. Download Table 3-1, DOC file.

Psychophysiological data were preprocessed and analyzed using MATLAB (version R2018b, MathWorks) with procedures implemented in PsPM 4.1.1 (Psychophysiological modeling, legacy version available at http://pspm.sourceforge.net), a MATLAB toolbox for model-based analysis of psychophysiological data (Bach and Friston, 2013; Bach et al., 2018a). For preprocessing of pupil data only, we used PsPM 5.1.0 (https://bachlab.github.io/PsPM/).

#### Data preprocessing and conditioned response scoring

##### Startle eye-blink responses

Preprocessing followed a peak-scoring procedure developed by Balderston et al. (2017) as implemented by Khemka et al. (2017). The raw EMG signal was high-pass filtered with a 4^th^ order Butterworth filter at 30 Hz, and an additional 50-Hz notch filter was used to remove mains noise. After rectification, data were smoothed with a 20-ms moving average. Preprocessed data were then averaged across all trials and visually inspected. Two participants (one doxycycline group, one placebo group) were excluded from analysis, because of missing average SEBR. To estimate conditioned responses, we recorded the maximum preprocessed EMG amplitude between 20 and 100 ms after startle sound onset, as determined from recording of the audio output. Two participants had no startle sound recordings. For these participants, we defined startle sound onset from the intended onset by adding the mean delay of startle sound onset in the other participants.

##### Skin conductance responses

To remove artefacts related to US presentation, all data points in the period from 0.2 s before US onset to 1.6 s after US onset were treated as missing values in all SCR analyses. Data were then visually inspected for remaining artefacts. One participant (doxycycline group) was excluded from all SCR analyses because of inadequate quality of the SCR signal but retained in all other analyses. SCR analysis was adapted from the procedure benchmarked in Staib et al. (2015): data were filtered with a 1st order unidirectional bandpass Butterworth filter (0.0159–5 Hz) and then downsampled to 10 Hz. This modification of filter settings had been validated in our previous methodological work (Wehrli et al., 2022). For conditioned response scoring, the standard nonlinear model implemented in PsPM was used. This provides trial-by-trial estimates of sudomotor bursts, which are modelled as Gaussian bump functions (Bach et al., 2010). As in the preceding methodological study, three bursts were modelled: two with constant latency in response to CS and US presentation, and one with estimated latency (but fixed dispersion) during the trace interval, between 10 s after CS offset and 1 s before US onset. All raw SCR amplitudes (before scaling, see below) were derived in μS, i.e., a neural input with unit amplitude would elicit an SCR with 1-μS amplitude.

##### Pupil size response

For conversion of EyeLink 1000 system’s arbitrary units to true diameter, we used the transformation derived in Hayes and Petrov (2016). Preprocessing followed the procedure by Kret and Sjak-Shie (2019) as implemented in PsPM 5.1.0. This procedure identifies valid samples by range, speed, edge, trendline and isolated sample filtering. Data were smoothed by filtering, interpolation and combined across both eyes. Intervals during which gaze direction was outside ±5° visual angle of the center of the screen were treated as missing. One participant (doxycycline group) had >50% missing data during the CS-US interval and was excluded from pupil size analysis.

Trial-by-trial pupil response was then estimated using the general linear convolution models (GLMs) approach implemented in PsPM (Korn et al., 2017), using a canonical response function specific to trace conditioning as derived in our previous methodological work (Wehrli et al., 2022).

#### Statistical analysis

##### Preregistered analysis

Statistical Analysis was performed in R (www.r-project.org), version 4.0.2. For trial-by-trial responses in SEBR, PSR, and SCR analysis, each participant’s amplitude estimate was normalized by dividing through the mean values in this participant’s CS− trials (Bach et al., 2018b, 2019). When inspecting the recall data for SCR visually after normalization, we found that response amplitude estimates on a small number of trials were implausibly high. We excluded individual trials with amplitude estimates outside of four standard deviations of the condition mean per trial over both groups. In total, 40 out of 20,160 trials in the placebo group and 47 out of 20,160 trials in the doxycycline group were excluded. No additional participants were removed, as none missed 50% or more of their trial data.

Acquisition data for SCR and PSR were analyzed with a preregistered 2 (group) × 2 (condition, i.e., CS+/CS−) × 40 (trial) linear mixed effects (LME) model using the R package “lmerTest” (version 3.1.2) function lmer() with trial number as a linear predictor across conditions. The trial numbers are represented as across trials to reflect that CS presentation is randomized and SCR estimates habituate over time (rather than just within conditions). This leads to an unbalanced model which is amenable to the LME approach. For consistency with previous work, we also averaged response estimates from placebo participants across all CS+ and CS− trials separately and computed a paired *t* test for the CS+/CS− difference. Significant results were Holm–Bonferroni corrected for four comparisons (i.e., SCR CS time point, SCR trace interval, SCR US time point and PSR) using the p.adjust() function of the “stats” package version 4.0.2.

Our preregistered primary outcome was SEBR data from the recall session, averaged across CS+ and CS− trials separately, and compared in a two-sample *t* test for the CS+/CS− difference. For consistency with previous work, we also tested the CS+/CS− difference within the placebo group. Second, because SEBR habituate over time, regardless of extinction, we further tested for group differences with a preregistered 2 (group) × 2 (condition) × 15 (trial) repeated measures ANOVA. For this we used the function aov() of the R “stats” package version 4.0.2, with trial indicating the trial index within the condition.

Our preregistered secondary outcome was SCR from the recall session. To account for time effects, these were analyzed in a 2 (group) × 2 (condition) × 30 (trial) LME model. Again, using the function lmer() of the R package “lmerTest” (version 3.1.2) with trial number as a function of time across conditions. Significant results were Holm–Bonferroni corrected for three comparisons (i.e., SCR CS time point, SCR trace interval and SCR US time point).

For all LMEs, we tested different random effect structures and retained the model with lowest Akaike’s information criterion (AIC) using the “stats” package (version 4.0.2) function AIC(). In case of nonconvergence with the default optimizer, we tested convergence with all available optimizers using the allFit() function of the “lme4” package (version 1.1.23). If models did not converge with alternate optimizers, the respective random effect structure was not considered further. Following this procedure, for all data from the acquisition session and for SCR to the time point of expected US presentation in the recall session, we retained a model with a random intercept per subject. For the remaining analyses, models with random effects accounting for subject and trial were retained. For effect size estimation we used the function eta_squared() of the “effect size” package 0.6.0.1.

##### Robustness analyses

Primary analyses used different statistical models for the different measures. To make them comparable and check the robustness of findings, we conducted additional (not preregistered) analyses. For SEBR, we computed a 2 (group) × 2 (condition, i.e., CS+/CS−) × 20 (trial) LME model with trial number as a function of time across conditions. Additionally, for SCR to CS onset during acquisition, LME revealed a main effect group that was not apparent in the descriptive statistics. Hence, this analysis was repeated using the “nlme” package version 3.1.149 function lme().

##### Exploratory analysis

Because individuals might differ in their metabolization of doxycycline, we investigated the relation of doxycycline serum levels with SEBR and SCR within the doxycycline group. To this end, we replaced the drug factor in the ANOVA (SEBR) and LME (SCR) analysis with doxycycline level as a linear predictor.

Furthermore, during data analysis, we found differences in fear retention between the sexes. For this reason, we separately investigated fear retention in men and women for SEBR and SCR in a *post hoc* analysis. Furthermore, we compared serum levels of doxycycline between men and women and tested how well doxycycline levels can be predicted by sex and weight with a linear model using the function lm() of the R “stats” package 4.0.2. Additionally, we performed a mediation analysis using the function mediate() of the R package “mediation” version 4.5.0 to identify the effect of sex on doxycycline serum levels mediated by weight.

Finally, when comparing state anxiety scores (Laux et al., 1981), we found unexpected differences between the placebo and doxycycline group, which had not previously been identified (Bach et al., 2018b). To investigate this difference, we performed an exploratory 2 (group) × 2 (time point) repeated measures ANOVA and follow-up independent *t* tests.

### Code and data accessibility

Analysis code is available on OSF (https://osf.io/uqtr5/). The anonymized dataset is available on www.zenodo.org (DOI: 10.5281/zenodo.6594800).

## Results

### Contingency memory and subjective ratings

Participants in the placebo group reported a CS+/CS− difference in CS-US contingency after the acquisition session, as did participants in the doxycycline group (paired *t* tests, *p* < 0.05), with no evidence for a difference between groups. Both groups remembered the association until after the recall session (paired *t* test, *p* < 0.05) and learned the new CS+ contingency during the recall session (paired *t* test, <0.05, for CS+ acquisition vs recall), again with no evidence for group differences (see Fig. 2). Contingency estimates substantially deviated from the objective reinforcement rates. Furthermore, participants in both groups indicated more negative feelings toward the CS+ than the CS− and more arousal by the CS+ than the CS− (Table 2).

**Table 2:** *t* test for difference between CS+ and CS− in valence and arousal ratings

Valence	Session	*t*	df	*p*-value	Mean CS− (±SD)	Mean CS+ (±SD)
Placebo	Acquisition	7.81	47	<0.001*	72.56 ± 21.59	31.88 ± 20.80
	Recall	4.33	47	<0.001*	61.59 ± 22.49	43.29 ± 21.33
Doxycycline	Acquisition	6.34	48	<0.001*	74.11 ± 21.36	36.51 ± 23.85
	Recall	3.47	48	0.001*	56.56 ± 21.21	43.62 ± 20.91

Arousal	Session	*t*	df	*p*-value	Mean CS− (±SD)	Mean CS+ (±SD)

Placebo	Acquisition	−8.11	47	<0.001*	21.16 ± 21.75	69.25 ± 26.99
	Recall	−3.93	47	<0.001*	35.73 ± 27.18	53.33 ± 26.53
Doxycycline	Acquisition	−5.41	48	<0.001*	36.22 ± 30.44	67.97 ± 24.83
	Recall	−3.46	48	0.001*	34.48 ± 29.60	48.20 ± 30.30

Valence ratings: “How do you feel when seeing this triangle? (0 = very negative, 100 = very positive)” Arousal ratings: “How aroused do you feel when seeing this triangle? (0 = very calm, 100 = very excited).” *p*-value: paired *t*-test significant differences (*p* < 0.05) are marked with ‘*’; SD: standard deviation.

### Placebo group analysis

**Figure 4.**
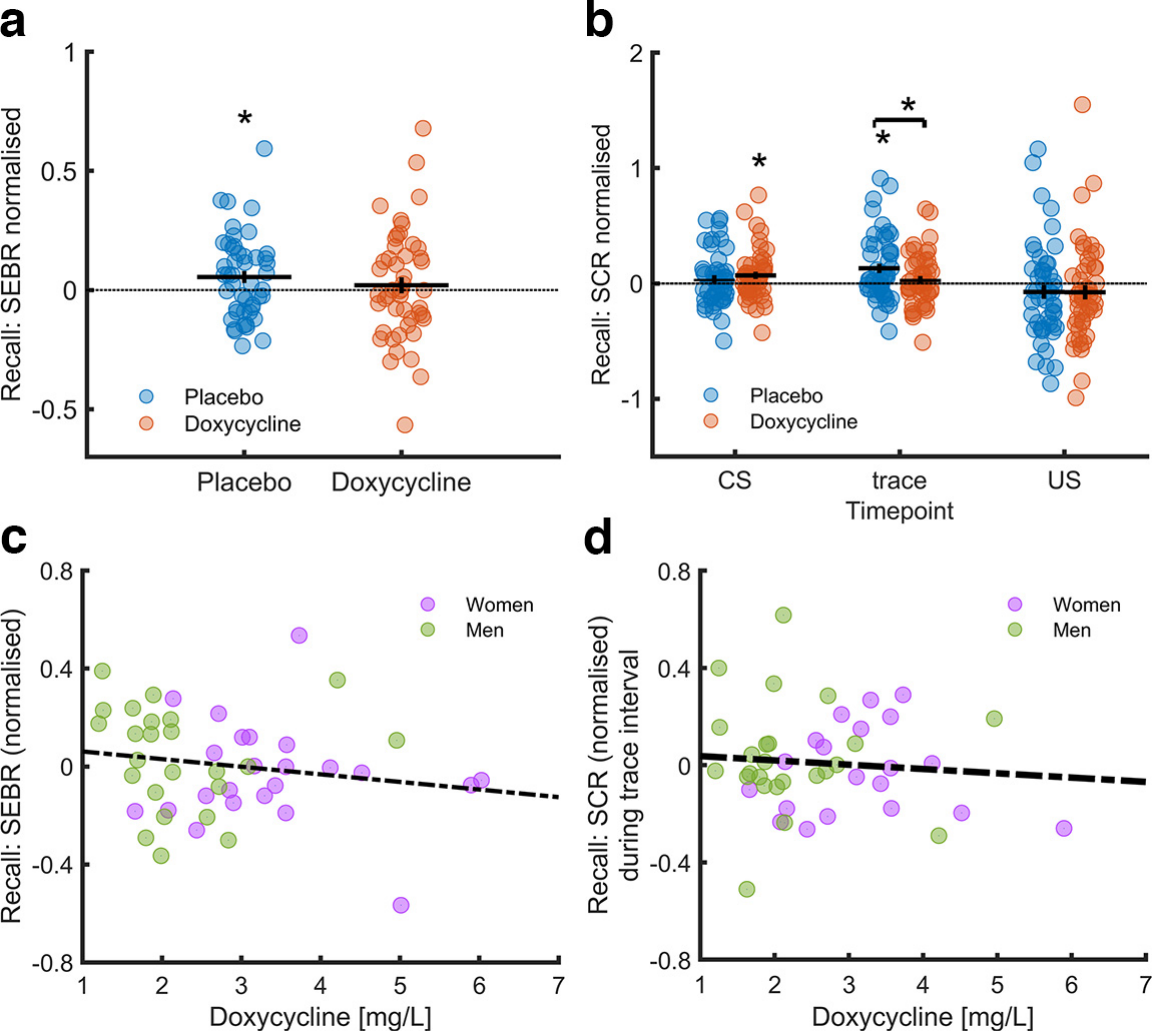
CS+/CS− differences in startle-eye blink responses (SEBR) and skin conductance responses (SCR) during recall visit 3 and their correlation with doxycycline level in serum. ***a***, CS+/CS− differences in SEBR during recall test. Normalized SEBR difference between CS+/CS− in the recall session, averaged over all trials. ***b***, CS+/CS− differences in SCR during recall test. Normalized SCR difference between CS+/CS− in the recall session averaged over all trials. Horizontal line: mean; vertical line: standard error of mean (SEM), asterisk denote significant (*p* < 0.05) difference between CS+ and CS− trials, asterisk above a line denote significant difference (*p* < 0.05 between placebo and doxycycline group). Individual levels of participants in the placebo group are depicted in blue, doxycycline group in red. For detailed analysis see Extended Data Figures 4-1, 4-2, 4-3, 4-4, 4-5, and 4-6. ***c***, Correlation of CS+/CS− difference in SEBR amplitudes in doxycycline group with the doxycycline concentration in serum after acquisition session visit 2, correlation of doxycycline level and SEBR amplitudes *r* = −0.17. ***d***, Correlation of CS+/CS− difference in SCR during trace interval in doxycycline group with the doxycycline concentration in serum after acquisition session visit 2, correlation of doxycycline level and SCR amplitudes *r* = −0.08. Individual levels of women are depicted in violet. Men are depicted in green. Dotted line shows regression regardless of sex. For more details see Table 5 and Extended Data Figure 4-7.

To control the effectiveness of the paradigm, we verified trace fear acquisition and retention within the placebo group. Averaged across the entire acquisition session, we found CS+/CS− differentiation for SCR to CS presentation (*t*_(47)_ = 4.06, *p* < 0.001, *d* = 0.59), SCR during the trace interval (*t*_(47)_ = 4.45, *p* < 0.001, *d* = 0.64), and for PSR (*t*_(47)_ = 7.81, *p* < 0.001, *d* = 1.13). In the recall test, SEBR differed between CS+/CS− (*t*_(46)_ = 2.14, *p* = 0.037, *d* = 0.31). These results indicate successful learning and memory retention in our paradigm (see Figs. 3, 4*a*; Extended Data Figs. 3-1, 4-1). As in our previous work (Wehrli et al., 2022), there was no evidence for a CS+/CS− difference in PSR in the recall test (see Extended Data Fig. 4-1).

**Table 5:** Relation of doxycycline levels with SEBR

	df	*F* value	*p*-value
Doxycycline level	1, 46	9.86	0.003*
Condition (CS+/CS−)	1, 1334	0.50	0.481
Trial number	14, 1334	21.62	<0.001*
Doxycycline level × Condition	1, 1334	11.54	<0.001*
Doxycycline level × Trial	14,1334	1.98	0.017*
Condition × Trial	14, 1334	0.61	0.856
Doxycycline level × Condition × Trial	14, 1334	2.93	<0.001*

ANOVA for the relation of doxycycline levels with SEBR, dependent on condition and trial number, only for the doxycycline group. Significant effects are marked with ‘*’.

10.1523/ENEURO.0243-22.2023.f4-1 Extended Data Figure 4-1 Extinction paired t test CS+/CS−, not corrected for multiple comparisons. Download Figure 4-1, DOC file.

10.1523/ENEURO.0243-22.2023.f4-2 Extended Data Figure 4-2 Extinction independent t test between CS+/CS− difference for placebo and doxycycline group, not corrected for multiple comparisons. P = Placebo, D = Doxycycline Download Figure 4-2, DOC file.

10.1523/ENEURO.0243-22.2023.f4-3 Extended Data Figure 4-3 Extinction paired t test CS+/CS− per gender, not corrected for multiple comparisons. Download Figure 4-3, DOC file.

10.1523/ENEURO.0243-22.2023.f4-4 Extended Data Figure 4-4 Extinction independent t test between CS+/CS− difference for placebo and doxycycline group per gender, not corrected for multiple comparisons. P = Placebo, D = Doxycycline Download Figure 4-4, DOC file.

**Figure 5.**
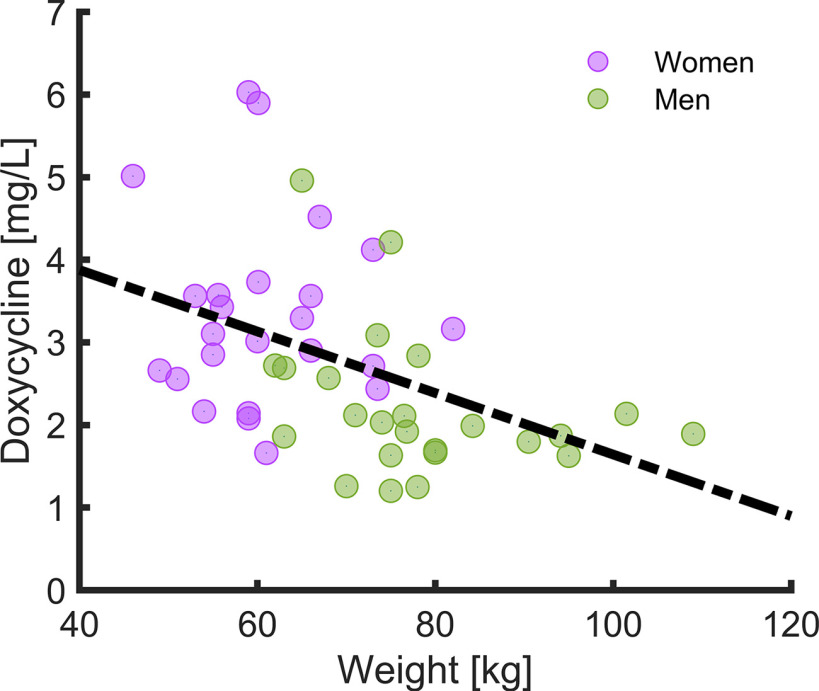
Correlation of body weight with doxycycline level in serum after acquisition session visit 2. Individual serum levels for women are depicted in violet. Men are depicted in green. Dotted line shows regression regardless of sex. For details, see Extended Data Figure 5-1.

10.1523/ENEURO.0243-22.2023.f4-5 Extended Data Figure 4-5 SEBR ANOVA in fear recall. Download Figure 4-5, DOC file.

**Figure 6.**
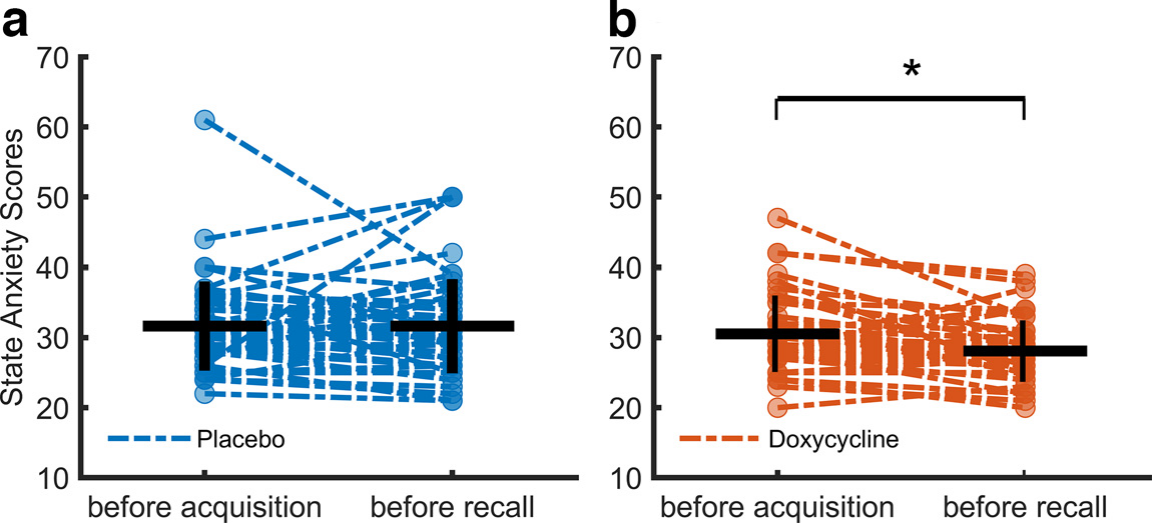
Anxiety ratings. ***a***, Placebo group, individual ratings depicted in blue. ***b***, Doxycycline group. Individual ratings depicted in red. Mean values are marked with a horizontal black line, standard deviation (SD) is depicted with a vertical line. Asterisk above a line denote significant difference (*p* < 0.05) between the anxiety ratings before acquisition and after recall.

10.1523/ENEURO.0243-22.2023.f4-6 Extended Data Figure 4-6 SEBR LME in fear recall. Download Figure 4-6, DOC file.

10.1523/ENEURO.0243-22.2023.f4-7 Extended Data Figure 4-7 SCR and doxycycline level (in doxycycline group) LME in fear recall. Download Figure 4-7, DOC file.

### Doxycycline and trace fear acquisition

Next, we investigated drug differences in a preregistered LME model for SCR and PSR. For SCR, we found higher CS+ than CS− responses at all time points (main effect CS), larger SCR to CS presentation in the doxycycline group (main effect group), and faster SCR habituation in the placebo group (group × trial interaction), to CS presentation and during the trace interval (see Table 3). There was no significant interaction of group and condition. For PSR, we found higher CS+ than CS− responses, and no impact of doxycycline (Table 3). For SCR to CS presentation, the main effect group was not replicated in the robustness analysis, whereas all other results were (see Extended Data Table 3-1).

**Table 3:** LME analysis of SCR and PSR during trace fear acquisition

	CS presentation			Trace interval			US time point		
Fear acquisition SCR	*F* value	df	*p*-value	*F* value	df	*p*-value	*F* value	df	*p*-value
Drug (doxycycline/placebo)	8.99	1, 1002.7	0.003*	0.43	1, 324.8	0.51	2.74	1, 266.2	0.1
Condition (CS+/CS−)	22.27	1, 3727.3	>0.001*	42.9	1, 3726	>0.001*	78.6	1, 3731.9	>0.001*
Trial number	16.97	1, 3726.2	>0.001*	11.2	1, 3725.1	>0.001*	27.2	1, 3731.2	>0.001*
Drug × Condition	0.61	1, 3727.3	0.435	3.68	1, 3726	0.06	3.74	1, 3731.9	0.05
Drug × Trial	11.79	1, 3726.2	>0.001*	7.23	1, 3725.1	0.007*	3.16	1, 3731.2	0.08
Condition × Trial	2.09	1, 3728.1	0.149	1.24	1, 3726.5	0.27	2.9	1, 3732.1	0.09
Drug × Condition × Trial	0.45	1, 3728.1	0.501	0.36	1, 3726.5	0.55	2.12	1, 3732.1	0.15

Fear acquisition PSR	*F* value	df	*p*-value						

Drug (doxycycline/placebo)	0.2	1, 484.9	0.655						
Condition (CS+/CS−)	53.5	1, 3739.4	>0.001*						
Trial number	0.75	1, 3738	0.387						
Drug × Condition	0.63	1, 3739.4	0.429						
Drug × Trial	0.06	1, 3738	0.803						
Condition × Trial	3.04	1, 3739.8	0.081						
Drug × Condition × Trial	1.56	1, 3739.8	0.212						

Models are estimated with lme4, lmer(data ∼ (1|subject) + group*condition*trial), significant (*p* < 0.05) effects are marked with a * after Holm–Bonferroni correction, for robustness analysis, see Extended Data Table 3-1.

In an exploratory analysis, we tested for sex differences and found that both men and women differentiate CS+/CS− successfully, both in the placebo and doxycycline group (see Extended Data Fig. 3-3), with no significant group differences (see Extended Data Fig. 3-4).

10.1523/ENEURO.0243-22.2023.f3-3 Extended Data Figure 3-3 Acquisition paired t test CS+/CS− per gender, not corrected for multiple comparisons. Download Figure 3-3, DOC file.

10.1523/ENEURO.0243-22.2023.f3-4 Extended Data Figure 3-4 Acquisition independent t test between CS+/CS− difference for placebo and doxycycline group per gender, not corrected for multiple comparisons. P = Placebo, D = Doxycycline Download Figure 3-4, DOC file.

### Trace fear memory recall: preregistered analyses

Our preregistered primary outcome measure for trace fear memory retention was fear-potentiated startle, quantified by SEBR. A direct group comparison, our preregistered primary analysis, revealed no significant difference between doxycycline and placebo (*t*_(87.464)_ = −0.83, *p* = 0.41, *d* = 0.17; Fig. 4*a*). Also, our preregistered secondary analysis, a 2 (group) × 2 (CS+/CS−) × 15 (trial) repeated measures ANOVA, revealed no effect of group, condition, or group × condition (see Extended Data Fig. 4-5). This result was confirmed in a robustness analysis using LME (see Extended Data Fig. 4-6).

Our preregistered secondary outcome measure of trace fear retention was SCR. In our preregistered LME analysis, we found a significant effect of trial, suggesting habituation. There were no other significant effects after Holm–Bonferroni correction for the three time points (see Table 4).

**Table 4:** LME analysis of SCR during trace fear recall

	CS presentation			Trace interval			US time point		
Fear recall SCR	*F* value	df	*p*-value	*F* value	df	*p*-value	*F* value	df	*p*-value
Drug (doxycycline/placebo)	0.53	1, 94.19	0.468	2.13	1, 89.52	0.148	1.50	1, 1173.40	0.220
Condition (CS+/CS−)	5.55	1, 2774.23	0.019	5.57	1, 2770.09	0.018	0.56	1, 2745.20	0.455
Trial number	13.55	1, 94.11	>0.001*	38.35	1, 89.51	>0.001*	23.77	1, 2743.00	>0.001*
Drug × Condition	0.02	1, 2774.23	0.889	1.66	1, 2770.09	0.198	0.10	1, 2745.20	0.749
Drug × Trial	0.72	1, 94.11	0.397	1.58	1, 89.51	0.212	1.86	1, 2743.00	0.173
Condition × Trial	2.18	1, 2774.86	0.140	0.86	1, 2771.01	0.353	0.00	1, 2746.80	0.999
Drug × Condition × Trial	0.01	1, 2774.86	0.920	0.11	1, 2771.01	0.737	0.15	1, 2746.80	0.699

Models are estimated with lme4, lmer(data ∼ (1+trial|subject) + group*condition*trial) for CS and US time point and lmer(data ∼ (1|subject) + group*condition*trial) for trace interval, significant (*p* < 0.05) effects are marked with a * after Holm–Bonferroni correction.

### Trace fear memory recall: exploratory analyses

A group comparison of CS+/CS− differences in SCR, averaged over the entire recall session, revealed a significant group difference during the trace interval (*t*_(89.01)_ = 2.02, *p* = 0.046, *d* = 0.41), but not to CS presentation (*t*_(92.87)_ = −0.60, *p* = 0.55, *d* = 0.12; see Fig. 4*b*; Extended Data Figs. 4-1 and 4-2). This difference during the trace interval resulted from the larger CS+/CS− differentiation in the placebo group (*t*_(47)_ = 3.19, *p* = 0.003, *d* = 0.46), as opposed to the doxycycline group (*t*_(47)_ = 0.78, *p* = 0.44, *d* = 0.11), indicating weaker memory retention in the doxycycline group.

Participants may have metabolized the study drug differently. We analyzed serum samples taken at the end of the study and investigated a relation of doxycycline serum level with SEBR and SCR during the trace interval within the doxycycline group (see Fig. 4*c*,*d*). We found a significant negative relation of doxycycline level with CS+/CS− differences in SEBR (see Table 5; Fig. 4*c*) but not in SCR during the trace interval (see Fig. 4*d*; Extended Data Fig. 4-7).

We incidentally observed apparent sex differences in fear-potentiated SEBR: within the placebo group, females showed memory retention (*t*_(22)_ = 4.12, *p* < 0.001, *d* = 0.86) while males did not (*t*_(23)_ = 0.40, *p* = 0.69, *d* = 0.08). This motivated analyzing the impact of doxycycline on females separately. Females in the doxycycline group showed no memory retention (*t*_(23)_ = 0.00, *p* = 0.10, *d* = 0.00). However, the placebo/drug difference in females was not significant (*t*_(31.44)_ = −1.66, *p* = 0.11, *d* = 0.48). Similarly, females in the placebo group showed memory retention in SCR during the trace interval (*t*_(23)_ = 2.58, *p* = 0.017, *d* = 0.53) and males did not (*t*_(23)_ = 1.91, *p* = 0.069, *d* = 0.39; see Extended Data Fig. 4-3). While females in the doxycycline group showed no memory retention (*t*_(23)_ = 0.37, *p* = 0.72, *d* = 0.08), the placebo/drug difference in females was not significant (*t*_(42.75)_ = 1.86, *p* = 0.073, *d* = 0.53; see Extended Data Fig. 4-4).

### Doxycycline serum levels

Doxycycline serum levels differed between females (mean ± SD: 3.34 ± 1.12 mg/l) and males (mean ± SD: 2.21 ± 0.89 mg/l; *t*_(46)_ = 3.86, *p* < 0.001, *d* = 1.11). After controlling for body weight (females: mean ± SD: 60.93 ± 7.80, males: mean ± SD: 78.36 ± 12.14; *t*_(81)_ = 7.67, *p* < 0.001, *d* = 1.69), we identified a relation between doxycycline concentration and sex (*t*_(45)_ = 2.15, *p* = 0.037, η^2^ = 0.25; see Fig. 5). In a mediation analysis with doxycycline concentration as outcome, weight as the mediator and sex as the independent variable, we found that 28% of the sex effect was modulated by weight (see Extended Data Fig. 5-1).

10.1523/ENEURO.0243-22.2023.f5-1 Extended Data Figure 5-1 Mediation analysis for effect of sex on doxycycline serum levels mediated by weight. Download Figure 5-1, DOC file.

### State anxiety

We recorded state anxiety using the STAI (Laux et al., 1981) immediately before the acquisition and recall sessions. An exploratory 2 (group) × 2 (time point) repeated measures ANOVA revealed an effect of group (*F* = 4.86, *p* = 0.030), time point (*F* = 4.12, *p* = 0.046) and an interaction of group × time point (*F* = 4.38, *p* = 0.039 on state anxiety; see Fig. 6). Follow-up *t* tests showed that the groups had comparable anxiety levels before acquisition (*t*_(91)_ = 0.47, *p* = 0.370, *d* = 0.19), but differed before the recall session (*t*_(91)_ = 3.00, *p* = 0.004, *d* = 0.62), because of a decrease in anxiety levels in the doxycycline group.

## Discussion

Previous work has identified the MMP9 inhibiting drug doxycycline as a possible inhibitor of human fear memory consolidation (Bach et al., 2018b). Such properties may have potential for clinical application in secondary prevention of fear and trauma-related disorders, such as posttraumatic stress disorder (Iyadurai et al., 2018). In the present work, we tested the impact of doxycycline on long-interval (15 s) trace fear conditioning, which models a temporal gap between cue and outcome as an important feature of real-life trauma and relies on a wider neural network including hippocampus (Gilmartin et al., 2012). Unexpectedly, our preregistered analyses revealed no evidence for trace fear memory attenuation by doxycycline, neither in our preregistered primary outcome, differential SEBR, nor in the secondary outcome, differential SCR. In the following, we discuss this finding again from a neurobiological, statistical, and methodological perspective.

First, a likely conclusion from our study is that doxycycline has a smaller than anticipated – or no – impact on trace fear conditioning. This null finding contrasts with previous work on delay fear conditioning (Bach et al., 2018b). Nonhuman experiments have provided evidence that trace fear conditioning relies on neural circuits that extend those involved in delay conditioning, in particular including hippocampal neurons (Gilmartin et al., 2012). It is not known to what extent synaptic plasticity in additional areas is required for trace fear conditioning, and whether this also involves MMP9 signaling. Hence, it is possible that MMP9 inhibition would have a lower impact on trace fear conditioning. In addition, pharmacokinetic factors could contribute to our null result. A *post hoc* exploratory test suggested that fear memory retention in the doxycycline group related to serum levels, indicating that the effect of doxycycline hinges on sufficient metabolization. Previous work on doxycycline impact on delay conditioning did not assess drug metabolization (Bach et al., 2018b). Because this is a *post hoc* analysis, replication in a larger sample would allow clearer conclusions. If confirmed, this might motivate strategies to improve drug uptake. Exploratory tests indicated that weight and sex related to doxycycline serum levels. Thus, future studies may consider adapting the doxycycline dosage to body weight and/or sex, to improve efficacy across participants. As a caveat, we measured doxycycline levels at the end of the experiments rather than at the moment when fear acquisition took place. Furthermore, serum-level of doxycycline does not directly reflect concentrations of doxycycline in the brain. Drug penetration of doxycycline into the CSF is suggested to be 0.2 CSF/serum (Nau et al., 2010).

Second, results from parallel methodological work and the current placebo group indicated that our preregistered and exploratory analyses were unexpectedly underpowered. Effect size to measure trace fear memory retention was smaller in the present placebo group (Cohen’s *d* = 0.31) than in two preceding experiments not involving drugs (Cohen’s *d* = 0.44; Wehrli et al., 2022). Furthermore, effect size identified in these previous experiments was much smaller than the effect size for delay fear conditioning in methodological studies (Cohen’s *d* = 1.17; Khemka et al., 2017). Because these previous studies were analyzed while recruitment for the present study was already ongoing, sample size for the current work was based on the assumption of the much larger effect size found in delay conditioning. *Post hoc*, given an effect size of Cohen’s *d* = 0.44, an at least 60% reduction in memory retention in the doxycycline group (corresponding to the effect magnitude reported by Bach et al. (2018b) would equate to an effect size of *d* = 0.26. Power to detect an effect of this or larger size in our sample was 36%. Thus, our statistical results should be interpreted with caution. Future studies might seek to improve statistical power in this paradigm, for example by reducing the duration of the trace interval, or by removing the ITI task.

Finally, we found no evidence that shortcomings in experimental methodology account for the null result. We verified that in keeping with our previous work (Wehrli et al., 2022), our paradigm induced trace fear acquisition in the placebo group, as well as trace fear memory recall after one week. Because doxycycline was administered before fear acquisition, it may potentially have influenced fear acquisition. However, both placebo and doxycycline group differentiated CS+/CS− in SCR and PSR during acquisition. While we did find a drug × trial interaction in SCR, this suggested faster SCR habituation in the placebo group and no specific impact on acquisition. A previous delay fear conditioning study (Bach et al., 2018b) reported larger CS+/CS− differentiation during acquisition in doxycycline versus placebo, which was not replicated here.

Our approach to treatment development was based on preventing fear memory consolidation; but there are also other strategies. A somewhat related approach is to interfere with the reconsolidation of already-consolidated memory which relies on a similar molecular signaling cascade (Dudai and Eisenberg, 2004; Alberini, 2005; Tronson and Taylor, 2007). An altogether different strategy is improvement of extinction learning, which forms the basis for many psychotherapy-based interventions. Enhancement of fear extinction with levodopa (L-DOPA) showed to be a promising accessory to exposure therapy (Gerlicher et al., 2019), although a later study found only a reduction of reinstatement but no improvement of extinction in recall in PTSD patients (Cisler et al., 2020).

To conclude, we found no evidence that doxycycline impacts on long-interval trace fear conditioning. Unexpectedly, effect sizes in our paradigm were generally low in several independent control samples, to the extent that replication studies with sufficient power might be impractical because of the required sample size (*N* = 368 participants for 80% statistical power under the best-case assumptions outlined above). For future studies assessing the impact of MMP9 inhibition, we suggest focusing on preclinical paradigms with higher statistical power that may allow clearer conclusions, on strategies to ensure comparable drug uptake across participants, or on alternative drugs with more consistent metabolization.

## Synthesis

Reviewing Editor: Ifat Levy, Yale University School of Medicine Decisions are customarily a result of the Reviewing Editor and the peer reviewers coming together and discussing their recommendations until a consensus is reached. When revisions are invited, a fact-based synthesis statement explaining their decision and outlining what is needed to prepare a revision will be listed below. The following reviewer(s) agreed to reveal their identity: NONE. Note: If this manuscript was transferred from JNeurosci and a decision was made to accept the manuscript without peer review, a brief statement to this effect will instead be what is listed below.

In this randomized-control study the matrix metalloproteinase (MMP)-9 inhibitor antibiotic doxycycline was used to test whether MMP-9 blockade reduces fear memory retention in Pavlovian trace fear conditioning. The study was pre-registered and all analyses were closed before unblinding groups relation. The main pre-registered outcome was fear potentiated startle (FPS) and secondary outcome were skin conductance response (SCR), pupil size (PSR), and respiratory amplitude in the interaction between drug and condition (CS+/CS-) during the retention phase. The main outcome was no drug by condition interaction or effect for drug on FPS. Secondary outcomes show no drug by condition interaction in any of the secondary measures and main effect for drug only in SCR. An analysis with trial showed an interaction between trial and group, taken together suggesting an overall lower response in the placebo group.

Major comment:

This study is well designed, pre-registered and according to the method section well powered. However, the main point does not seem justified. With all 4 measures (FPS, SCR, PSR, and respiration) failing to show a group by condition interaction during retention, the data suggest no evidence for MMP-9 reducing fear memory. As these were the pre-registered hypotheses, the authors should stick to them as the main story, and only after that enter into exploratory analysis of additional factors such as sex and anxiety.

Minor comments:

- Similarly to comments from the previous reviewers, the text still needs editing. There is misuse of tenses such as line 53 “we find”; missing parts of sentences, e.g. line 219 “During a 180-min metabolization interval”; Sentences over 40 words long; over- and misuse of acronyms (is SEBR different from FPS?) Line 194 “doxycycline is detectable in CSF, the cerebrospinal fluid (CSF)”. Titles of the physiological measures are acronyms such as the SEBR, or no new line for the SCR, which is written as SC.

- The methods should be streamlined. Currently, some parts are introduced in an order that makes it hard to understand. For example, the CS+, CS- and US are introduced, but the reinforcement rate is in a completely different segment, which makes it hard to understand the flow of the experiment. The methods lack details about how the responses were quantified. As this is a trace fear conditioning, what was the event the analysis was locked to, in the FPS, to the sound, in the SCR to the CS, the CS onset, but for the trace? Did you look for a peak? Or measure tonic response? What units did you measure (microsiemens)?

- The analysis plan is hard to follow. In all tests you started with a t-test and later moved to higher order analysis. Why is that? Start with ANOVA or LME and then go to the post hoc t-tests. In the current analysis there are dozens of results, if you try to correct the article for multiple tests you will likely find that most are nullified. Why are you using repeated measures ANOVA in the FPS and LME in the SCR? The r commands are mentioned (aov(), lme()) instead of the package you used (lme4; add citation).

- As this substance is given 3 and a half hours before fear memory is created, what are the clinical implications? Using a reconsolidation paradigm might be more clinically relevant.
