# Supplementary material for: Effect of the Matrix Metalloproteinase Inhibitor Doxycycline on Human Trace Fear Memory
Source: eNeuro. 2023 Feb 23;10(2):ENEURO.0243-22.2023. doi: 10.1523/ENEURO.0243-22.2023 (PMC9961363; doi:10.1523/ENEURO.0243-22.2023)
Supplement: Extended Data Figure 3-1 — Acquisition paired t test CS+/CS−, not corrected for multiple comparisons. Download Figure 3-1, DOC file. [file enu-eN-NRS-0243-22-s02.doc]

| **Figure 3-1** |  |  |  |  |  |  |  |  |  |  |
| --- | --- | --- | --- | --- | --- | --- | --- | --- | --- | --- |
| Acquisition paired t-test CS+/CS-, not corrected for multiple comparisons | | | |  |  |  |  |  |  |  |
|  |  |  |  |  |  |  |  |  |  |  |
|  |  |  |  |  |  |  |  |  |  |  |
|  |  |  |  |  |  |  |  |  | **Mean (± SD)** | |
| **Measure** | **Group** | **Specification** | **averaged** | **t-statistic** | **p** | **df** | **95% CI** | **cohen's d** | **CS+** | **CS-** |
| SCR DCM | Placebo | to CS presentation | trial 1-20 | 4.33 | < .001* | 47 | [0.09, 0.25] | 0.62 | 1.17 ± 0.27 | 1.00 ± 0.00 |
|  |  | during trace interval | " | 4.75 | < .001* | 47 | [0.25, 0.63] | 0.69 | 1.42 ± 0.66 | 0.98 ± 0.06 |
|  |  | to US presentation | " | 5.32 | < .001* | 47 | [0.40, 0.89] | 0.77 | 1.64 ± 0.85 | 0.99 ± 0.04 |
|  | Doxycycline | to CS presentation | trial 1-20 | 5.21 | < .001* | 47 | [0.12, 0.26] | 0.75 | 1.17 ± 0.26 | 0.98 ± 0.07 |
|  |  | during trace interval | " | 7.13 | < .001* | 47 | [0.46, 0.83] | 1.03 | 1.63 ± 0.63 | 0.99 ± 0.06 |
|  |  | to US presentation | " | 4.65 | < .001* | 47 | [0.31, 0.77] | 0.67 | 1.53 ± 0.80 | 0.99 ± 0.05 |
| PSR | Placebo | fitted | trial 1-20 | 7.81 | < .001* | 47 | [0.19, 0.32] | 1.13 | 1.26 ± 0.23 | 1.00 ± 0.00 |
|  | Doxycycline | " | " | 8.48 | < .001* | 47 | [0.20, 0.33] | 1.22 | 1.27 ± 0.22 | 1.00 ± 0.00 |
| HP | Placebo | fitted | condition-wise | -0.71 | 0.48 | 47 | [-13.35, 6.37] | -0.10 | -33.11 ± 33.23 | -29.62 ± 32.29 |
|  | Doxycycline | " | " | 1.29 | 0.20 | 48 | [-2.69, 12.32] | 0.18 | -35.84 ± 34.67 | -40.66 ± 29.37 |
| RA | Placebo | early and late RF | condition-wise | -0.63 | 0.53 | 47 | [-0.26, 0.13] | -0.09 | -0.30 ± 0.48 | -0.24 ± 0.62 |
|  | Doxycycline | " | " | -1.24 | 0.22 | 48 | [-0.37, 0.09] | -0.18 | -0.43 ± 0.81 | -0.29 ± 0.72 |
