# Supplementary material for: Effect of the Matrix Metalloproteinase Inhibitor Doxycycline on Human Trace Fear Memory
Source: eNeuro. 2023 Feb 23;10(2):ENEURO.0243-22.2023. doi: 10.1523/ENEURO.0243-22.2023 (PMC9961363; doi:10.1523/ENEURO.0243-22.2023)
Supplement: Extended Data Figure 3-2 — Acquisition independent t test between CS+/CS− difference for placebo and doxycycline group, not corrected for multiple comparisons. P = Placebo, D = Doxycycline Download Figure 3-2, DOC file. [file enu-eN-NRS-0243-22-s03.doc]

| **Figure 3-2** |  |  |  |  |  |  |  |  |  |  |
| --- | --- | --- | --- | --- | --- | --- | --- | --- | --- | --- |
| Acquisition independent t-test between CS+/CS- difference for placebo and doxycycline group, not corrected for multiple comparisons | | | | | | | |  |  |  |
| P = Placebo, D = Doxycycline | |  |  |  |  |  |  |  |  |  |
|  |  |  |  |  |  |  |  |  |  |  |
|  |  |  |  |  |  |  |  |  | **Mean CSplus - CSminus (± SD)** | |
| **Measure** | **Group** | **Specification** | **averaged** | **t-statistic** | **p** | **df** | **95% CI** | **cohen's d** | **Placebo** | **Doxycycline** |
| SCR | P vs. D | to CS presentation | trial 1-20 | -0.35 | 0.72 | 93.38 | [-0.13, 0.09] | 0.07 | 0.17 ± 0.27 | 0.19 ± 0.25 |
|  |  | during trace interval | " | -1.59 | 0.11 | 93.96 | [-0.46, 0.05] | 0.32 | 0.44 ± 0.64 | 0.65 ± 0.63 |
|  |  | to US presentation | " | 0.63 | 0.53 | 93.83 | [-0.23, 0.44] | 0.13 | 0.65 ± 0.84 | 0.54 ± 0.81 |
| PSR | P vs. D | fitted | trial 1-20 | -0.16 | 0.87 | 93.73 | [-0.10, 0.08] | 0.03 | 0.26 ± 0.23 | 0.27 ± 0.22 |
| HP | P vs. D | fitted | trial 1-20 | -1.35 | 0.18 | 95 | [-20.50, 3.89] | -0.28 | -3.49 ± 33.94 | 4.81 ± 26.11 |
| RA | P vs. D | early and late RF | trial 1-20 | 0.52 | 0.60 | 95 | [-0.22, 0.37] | 0.11 | -0.06 ± 0.67 | -0.14 ± 0.79 |
