# Supplementary material for: Effect of the Matrix Metalloproteinase Inhibitor Doxycycline on Human Trace Fear Memory
Source: eNeuro. 2023 Feb 23;10(2):ENEURO.0243-22.2023. doi: 10.1523/ENEURO.0243-22.2023 (PMC9961363; doi:10.1523/ENEURO.0243-22.2023)
Supplement: Extended Data Table 3-1 — SCR to CS LME (estimated with “nlme” package) in fear acquisition. Download Table 3-1, DOC file. [file enu-eN-NRS-0243-22-s14.doc]

| **Table 3-1** |  |  |  |  |
| --- | --- | --- | --- | --- |
| SCR to CS LME (estimated with "nlme" package) in fear acquisition | | | | |
|  |  |  |  |  |
| **Fear recall SCR** | **F-value** | **df** | | **p-value** |
| Drug (doxycycline/placebo) | 0.02 | 1, | 94 | 0.88 |
| Condition (CS+/CS-) | 49.22 | 1, | 3726 | <0.001 |
| Trial number | 17.15 | 1, | 3726 | <0.001 |
| Drug x Condition | 0.17 | 1, | 3726 | 0.68 |
| Drug x Trial | 11.80 | 1, | 3726 | <0.001 |
| Condition x Trial | 2.08 | 1, | 3726 | 0.15 |
| Drug x Condition x Trial | 0.45 | 1, | 3726 | 0.50 |
