# Supplementary material for: Effect of the Matrix Metalloproteinase Inhibitor Doxycycline on Human Trace Fear Memory
Source: eNeuro. 2023 Feb 23;10(2):ENEURO.0243-22.2023. doi: 10.1523/ENEURO.0243-22.2023 (PMC9961363; doi:10.1523/ENEURO.0243-22.2023)
Supplement: Extended Data Figure 4-1 — Extinction paired t test CS+/CS−, not corrected for multiple comparisons. Download Figure 4-1, DOC file. [file enu-eN-NRS-0243-22-s06.doc]

| **Figure 4-1** |  |  |  |  |  |  |  |  |  |  |
| --- | --- | --- | --- | --- | --- | --- | --- | --- | --- | --- |
| Extinction paired t-test CS+/CS-, not corrected for multiple comparisons | | | |  |  |  |  |  |  |  |
|  |  |  |  |  |  |  |  |  |  |  |
|  |  |  |  |  |  |  |  |  | **Mean (± SD)** | |
| **Measure** | **Group** | **Specification** | **averaged** | **t-statistic** | **p** | **df** | **95% CI** | **cohen's d** | **CS+** | **CS-** |
| SEBR | Placebo | peak scoring | trial 1-15 | 2.14 | 0.037* | 46 | [0.00, 0.11] | 0.31 | 1.05 ± 0.17 | 1.00 ± 0.00 |
|  | Doxycycline | " | " | 0.60 | 0.55 | 47 | [-0.05, 0.09] | 0.09 | 1.02 ± 0.23 | 1.00 ± 0.00 |
| SCR DCM | Placebo | to CS presentation | trial 1-15 | 1.04 | 0.30 | 47 | [-0.03, 0.11] | 0.15 | 1.04 ± 0.24 | 1.00 ± 0.00 |
|  |  | during trace interval | " | 3.19 | 0.003* | 47 | [0.05, 0.21] | 0.46 | 1.11 ± 0.27 | 0.98 ± 0.09 |
|  |  | to US presentation | " | -1.26 | 0.21 | 47 | [-0.19, 0.04] | 0.18 | 0.84 ± 0.39 | 0.92 ± 0.24 |
|  | Doxycycline | to CS presentation | " | 2.07 | 0.044* | 47 | [0.00, 0.13] | 0.30 | 1.06 ± 0.22 | 1.00 ± 0.00 |
|  |  | during trace interval | " | 0.78 | 0.44 | 47 | [-0.04, 0.09] | 0.11 | 1.02 ± 0.21 | 1.00 ± 0.03 |
|  |  | to US presentation | " | -1.21 | 0.23 | 47 | [-0.20, 0.05] | 0.17 | 0.84 ± 0.44 | 0.91 ± 0.23 |
| PSR | Placebo | fitted | trial 1-15 | 1.83 | 0.07 | 47 | [0.00, 0.06] | 0.26 | 1.03 ± 0.12 | 1.00 ± 0.00 |
|  | Doxycycline | " | " | 0.98 | 0.33 | 47 | [-0.02, 0.05] | 0.14 | 1.02 ± 0.13 | 1.00 ± 0.00 |
