# Supplementary material for: Effect of the Matrix Metalloproteinase Inhibitor Doxycycline on Human Trace Fear Memory
Source: eNeuro. 2023 Feb 23;10(2):ENEURO.0243-22.2023. doi: 10.1523/ENEURO.0243-22.2023 (PMC9961363; doi:10.1523/ENEURO.0243-22.2023)
Supplement: Extended Data Figure 4-2 — Extinction independent t test between CS+/CS− difference for placebo and doxycycline group, not corrected for multiple comparisons. P = Placebo, D = Doxycycline Download Figure 4-2, DOC file. [file enu-eN-NRS-0243-22-s07.doc]

| **Figure 4-2** |  |  |  |  |  |  |  |  |  |  |
| --- | --- | --- | --- | --- | --- | --- | --- | --- | --- | --- |
| Extinction independent t-test between CS+/CS- difference for placebo and doxycycline group, not corrected for multiple comparisons | | | | | | | |  |  |  |
| P = Placebo, D = Doxycycline | |  |  |  |  |  |  |  |  |  |
|  |  |  |  |  |  |  |  |  |  |  |
|  |  |  |  |  |  |  |  |  | **Mean CSplus - CSminus (± SD)** | |
| **Measure** | **Group** | **Specification** | **averaged** | **t-statistic** | **p** | **df** | **95% CI** | **cohen's d** | **Placebo** | **Doxycycline** |
| SEBR | P vs. D | peak scoring | trial 1-15 | -0.83 | 0.41 | 87 | [-0.12, 0.05] | 0.17 | 0.05 ± 0.17 | 0.02 ± 0.23 |
| SCR | P vs. D | to CS presentation | trial 1-15 | -0.60 | 0.55 | 93 | [-0.12, 0.07] | 0.12 | 0.04 ± 0.24 | 0.06 ± 0.22 |
|  |  | during trace interval | " | 2.02 | 0.046* | 89 | [0.00, 0.20] | 0.41 | 0.13 ± 0.27 | 0.02 ± 0.22 |
|  |  | to US presentation | " | 0.02 | 0.98 | 94 | [-0.17, 0.17] | 0.00 | -0.07 ± 0.40 | -0.07 ± 0.43 |
| PSR | P vs. D | fitted | trial 1-15 | 0.52 | 0.60 | 93 | [-0.04, 0.06] | 0.11 | 0.03 ± 0.12 | 0.02 ± 0.13 |
