# Supplementary material for: Effect of the Matrix Metalloproteinase Inhibitor Doxycycline on Human Trace Fear Memory
Source: eNeuro. 2023 Feb 23;10(2):ENEURO.0243-22.2023. doi: 10.1523/ENEURO.0243-22.2023 (PMC9961363; doi:10.1523/ENEURO.0243-22.2023)
Supplement: Extended Data Figure 4-3 — Extinction paired t test CS+/CS− per gender, not corrected for multiple comparisons. Download Figure 4-3, DOC file. [file enu-eN-NRS-0243-22-s08.doc]

| **Figure 4-3** | |  |  |  |  |  |  |  |  |  |  |
| --- | --- | --- | --- | --- | --- | --- | --- | --- | --- | --- | --- |
| Extinction paired t-test CS+/CS- per gender, not corrected for multiple comparisons | | | | | |  |  |  |  |  |  |
|  |  |  |  |  |  |  |  |  |  |  |  |
|  |  |  |  |  |  |  |  |  |  | **Mean (± SD)** | |
| **Measure** | **Group** | **Gender** | **Specification** | **averaged** | **t-statistic** | **p** | **df** | **95% CI** | **cohen's d** | **CS+** | **CS-** |
| SEBR | Placebo | Women | peak scoring | trial 1-15 | 4.12 | <0.001* | 22 | [0.05, 0.14] | 0.86 | 1.09 ± 0.11 | 1.00 ± 0.00 |
|  |  | Men | " | " | 0.40 | 0.69 | 23 | [-0.07, 0.11] | 0.08 | 1.02 ± 0.22 | 1.00 ± 0.00 |
|  | Doxycycline | Women | " | " | 0.00 | 1.00 | 23 | [-0.11, 0.11] | 0.00 | 1.00 ± 0.25 | 1.00 ± 0.00 |
|  |  | Men | " | " | 0.93 | 0.36 | 23 | [-0.05, 0.13] | 0.19 | 1.04 ± 0.21 | 1.00 ± 0.00 |
| SCR DCM | Placebo | Women | to CS presentation | trial 1-15 | 1.70 | 0.10 | 23 | [-0.02, 0.17] | 0.35 | 1.08 ± 0.22 | 1.00 ± 0.00 |
|  |  |  | during trace interval | " | 2.58 | 0.017* | 23 | [0.03, 0.26] | 0.53 | 1.12 ± 0.29 | 0.98 ± 0.07 |
|  |  |  | to US presentation | " | -2.97 | 0.007* | 23 | [-0.35, -0.06] | 0.61 | 0.72 ± 0.33 | 0.93 ± 0.22 |
|  |  | Men | to CS presentation | " | -0.06 | 0.95 | 23 | [-0.11, 0.11] | 0.01 | 1.00 ± 0.26 | 1.00 ± 0.00 |
|  |  |  | during trace interval | " | 1.91 | 0.069 | 23 | [-0.01, 0.23] | 0.39 | 1.09 ± 0.25 | 0.98 ± 0.11 |
|  |  |  | to US presentation | " | 0.73 | 0.47 | 23 | [-0.11, 0.24] | 0.15 | 0.97 ± 0.41 | 0.91 ± 0.26 |
|  | Doxycycline | Women | to CS presentation | " | 1.99 | 0.059 | 23 | [0.00, 0.22] | 0.41 | 1.11 ± 0.27 | 1.00 ± 0.00 |
|  |  |  | during trace interval | " | 0.37 | 0.72 | 23 | [-0.07, 0.10] | 0.08 | 1.01 ± 0.19 | 0.99 ± 0.05 |
|  |  |  | to US presentation | " | -2.06 | 0.051 | 23 | [-0.28, 0.00] | 0.42 | 0.78 ± 0.36 | 0.92 ± 0.24 |
|  |  | Men | to CS presentation | " | 0.72 | 0.48 | 23 | [-0.04, 0.08] | 0.15 | 1.02 ± 0.15 | 1.00 ± 0.00 |
|  |  |  | during trace interval | " | 0.71 | 0.49 | 23 | [-0.06, 0.13] | 0.14 | 1.03 ± 0.23 | 1.00 ± 0.00 |
|  |  |  | to US presentation | " | -0.10 | 0.92 | 23 | [-0.22, 0.20] | 0.02 | 0.89 ± 0.50 | 0.90 ± 0.23 |
