# Supplementary material for: Effect of the Matrix Metalloproteinase Inhibitor Doxycycline on Human Trace Fear Memory
Source: eNeuro. 2023 Feb 23;10(2):ENEURO.0243-22.2023. doi: 10.1523/ENEURO.0243-22.2023 (PMC9961363; doi:10.1523/ENEURO.0243-22.2023)
Supplement: Extended Data Figure 4-4 — Extinction independent t test between CS+/CS− difference for placebo and doxycycline group per gender, not corrected for multiple comparisons. P = Placebo, D = Doxycycline Download Figure 4-4, DOC file. [file enu-eN-NRS-0243-22-s09.doc]

| **Figure 4-4** | |  |  |  |  |  |  |  |  |  |  |
| --- | --- | --- | --- | --- | --- | --- | --- | --- | --- | --- | --- |
| Extinction independent t-test between CS+/CS- difference for placebo and doxycycline group per gender, not corrected for multiple comparisons | | | | | | | | |  |  |  |
| P = Placebo, D = Doxycycline | |  |  |  |  |  |  |  |  |  |  |
|  |  |  |  |  |  |  |  |  |  |  |  |
|  |  |  |  |  |  |  |  |  |  | **Mean CSplus - CSminus (± SD)** | |
| **Measure** | **Group** | **Gender** | **Specification** | **averaged** | **t-statistic** | **p** | **df** | **95% CI** | **cohen's d** | **Placebo** | **Doxycycline** |
| SEBR | P vs. D | Women | peak scoring | trial 1-15 | -1.66 | 0.11 | 31.44 | [-0.21, 0.02] | 0.48 | 0.09 ± 0.11 | 0.00 ± 0.25 |
|  | P vs. D | Men | " | " | 0.36 | 0.72 | 45.96 | [-0.10, 0.15] | 0.10 | 0.02 ± 0.22 | 0.04 ± 0.21 |
| SCR | P vs. D | Women | to CS presentation | trial 1-15 | -0.44 | 0.66 | 44.49 | [-0.17, 0.11] | 0.13 | 0.08 ± 0.22 | 0.11 ± 0.27 |
|  |  |  | during trace interval | " | 1.84 | 0.073 | 42.75 | [-0.01, 0.27] | 0.53 | 0.14 ± 0.27 | 0.02 ± 0.20 |
|  |  |  | to US presentation | " | -0.71 | 0.48 | 45.95 | [-0.26, 0.13] | 0.20 | -0.21 ± 0.34 | -0.14 ± 0.33 |
|  | P vs. D | Men | to CS presentation | " | -0.41 | 0.68 | 36.44 | [-0.15, 0.10] | 0.12 | 0.00 ± 0.26 | 0.02 ± 0.15 |
|  |  |  | during trace interval | " | 1.03 | 0.31 | 44.20 | [-0.07, 0.23] | 0.30 | 0.11 ± 0.28 | 0.03 ± 0.23 |
|  |  |  | to US presentation | " | 0.54 | 0.59 | 44.25 | [-0.20, 0.34] | 0.16 | 0.06 ± 0.41 | -0.01 ± 0.50 |
