# Supplementary material for: Effect of the Matrix Metalloproteinase Inhibitor Doxycycline on Human Trace Fear Memory
Source: eNeuro. 2023 Feb 23;10(2):ENEURO.0243-22.2023. doi: 10.1523/ENEURO.0243-22.2023 (PMC9961363; doi:10.1523/ENEURO.0243-22.2023)
Supplement: Extended Data Figure 4-5 — SEBR ANOVA in fear recall. Download Figure 4-5, DOC file. [file enu-eN-NRS-0243-22-s10.doc]

| **Figure 4-5** |  |  |  |  |
| --- | --- | --- | --- | --- |
| SEBR ANOVA in fear recall |  |  |  |  |
|  |  |  |  |  |
| **Fear recall SEBR** | **F-value** | **df** | | **p-value** |
| Drug (doxycycline/placebo) | 0.68 | 1, | 93 | 0.41 |
| Condition (CS+/CS-) | 3.32 | 1, | 2697 | 0.069 |
| Trial number | 41.10 | 14, | 2697 | <0.001 |
| Drug x Condition | 0.73 | 1, | 2697 | 0.39 |
| Drug x Trial | 0.50 | 14, | 2697 | 0.94 |
| Condition x Trial | 1.11 | 14, | 2697 | 0.35 |
| Drug x Condition x Trial | 0.32 | 14, | 2697 | 0.99 |
