# Supplementary material for: Effect of the Matrix Metalloproteinase Inhibitor Doxycycline on Human Trace Fear Memory
Source: eNeuro. 2023 Feb 23;10(2):ENEURO.0243-22.2023. doi: 10.1523/ENEURO.0243-22.2023 (PMC9961363; doi:10.1523/ENEURO.0243-22.2023)
Supplement: Extended Data Figure 4-7 — SCR and doxycycline level (in doxycycline group) LME in fear recall. Download Figure 4-7, DOC file. [file enu-eN-NRS-0243-22-s12.doc]

| **Figure 4-7** |  |  |  |  |  |  |  |  |  |  |  |  |
| --- | --- | --- | --- | --- | --- | --- | --- | --- | --- | --- | --- | --- |
| SCR and doxycycline level (in doxycycline group) LME in fear recall | | | | |  |  |  |  |  |  |  |  |
|  |  |  |  |  |  |  |  |  |  |  |  |  |
|  | **to CS presentation** | |  |  | **Trace Interval** | |  |  | **US timepoint** | |  |  |
| **Fear recall SCR** | **F-value** | **df** | | **p-value** | **F-value** | **df** | | **p-value** | **F-value** | **df** | | **p-value** |
| Doxycycline level | 0.40 | 1, | 44.85 | 0.53 | 0.38 | 1, | 58.09 | 0.54 | 2.41 | 1, | 489.26 | 0.12 |
| Condition (CS+/CS-) | 0.05 | 1, | 1355.39 | 0.82 | 1.03 | 1, | 1350.60 | 0.31 | 0.45 | 1, | 1338.23 | 0.50 |
| Trial number | 0.00 | 1, | 44.89 | 0.95 | 1.02 | 1, | 57.12 | 0.32 | 12.24 | 1, | 1340.26 | <0.001* |
| Dox x Condition | 0.35 | 1, | 1355.52 | 0.55 | 0.66 | 1, | 1352.19 | 0.42 | 0.19 | 1, | 1337.22 | 0.67 |
| Dox x Trial | 0.58 | 1, | 44.89 | 0.45 | 0.42 | 1, | 57.27 | 0.52 | 3.59 | 1, | 1338.24 | 0.06 |
| Condition x Trial | 0.24 | 1, | 1355.88 | 0.62 | 0.82 | 1, | 1351.04 | 0.37 | 0.95 | 1, | 1339.14 | 0.33 |
| Dox x Condition x Trial | 1.08 | 1, | 1356.13 | 0.30 | 0.66 | 1, | 1352.66 | 0.42 | 0.90 | 1, | 1337.91 | 0.34 |
