# Supplementary material for: Effect of the Matrix Metalloproteinase Inhibitor Doxycycline on Human Trace Fear Memory
Source: eNeuro. 2023 Feb 23;10(2):ENEURO.0243-22.2023. doi: 10.1523/ENEURO.0243-22.2023 (PMC9961363; doi:10.1523/ENEURO.0243-22.2023)
Supplement: Extended Data Figure 3-3 — Acquisition paired t test CS+/CS− per gender, not corrected for multiple comparisons. Download Figure 3-3, DOC file. [file enu-eN-NRS-0243-22-s04.doc]

| **Figure 3-3** | |  |  |  |  |  |  |  |  |  |  |
| --- | --- | --- | --- | --- | --- | --- | --- | --- | --- | --- | --- |
| Acquisition paired t-test CS+/CS- per gender, not corrected for multiple comparisons | | | | |  |  |  |  |  |  |  |
|  |  |  |  |  |  |  |  |  |  |  |  |
|  |  |  |  |  |  |  |  |  |  | **Mean (± SD)** | |
| **Measure** | **Group** | **Gender** | **Specification** | **averaged** | **t-statistic** | **p** | **df** | **95% CI** | **cohen's d** | **CS+** | **CS-** |
| SCR DCM | Placebo | Women | to CS presentation | trial 1-20 | 3.21 | 0.004* | 23 | [0.08, 0.36] | 0.66 | 1.22 ± 0.33 | 1.00 ± 0.00 |
|  |  |  | during trace interval | " | 2.91 | 0.008* | 23 | [0.10, 0.60] | 0.59 | 1.35 ± 0.59 | 1.00 ± 0.00 |
|  |  |  | to US presentation | " | 3.34 | 0.003* | 23 | [0.25, 1.06] | 0.68 | 1.66 ± 0.96 | 1.00 ± 0.00 |
|  |  | Men | to CS presentation | " | 3.15 | 0.005* | 23 | [0.04, 0.20] | 0.64 | 1.12 ± 0.19 | 1.00 ± 0.00 |
|  |  |  | during trace interval | " | 3.76 | 0.001* | 23 | [0.24, 0.82] | 0.77 | 1.49 ± 0.74 | 0.96 ± 0.09 |
|  |  |  | to US presentation | " | 4.32 | < .001* | 23 | [0.33, 0.94] | 0.88 | 1.63 ± 0.73 | 0.99 ± 0.05 |
|  | Doxycycline | Women | to CS presentation | " | 2.92 | 0.008* | 23 | [0.04, 0.24] | 0.60 | 1.11 ± 0.25 | 0.97 ± 0.10 |
|  |  |  | during trace interval | " | 4.40 | < .001* | 23 | [0.32, 0.88] | 0.90 | 1.59 ± 0.67 | 0.99 ± 0.05 |
|  |  |  | to US presentation | " | 4.42 | < .001* | 23 | [0.33, 0.91] | 0.90 | 1.61 ± 0.67 | 0.99 ± 0.05 |
|  |  | Men | to CS presentation | " | 4.44 | < .001* | 23 | [0.13, 0.35] | 0.91 | 1.24 ± 0.26 | 1.00 ± 0.00 |
|  |  |  | during trace interval | " | 5.68 | < .001* | 23 | [0.44, 0.95] | 1.16 | 1.68 ± 0.59 | 0.98 ± 0.08 |
|  |  |  | to US presentation | " | 2.47 | 0.024* | 23 | [0.85, 0.50] | 0.50 | 1.45 ± 0.92 | 0.99 ± 0.05 |
| PSR | Placebo | Women | fitted | trial 1-20 | 5.23 | < .001* | 23 | [0.16, 0.38] | 1.07 | 1.27 ± 0.26 | 1.00 ± 0.00 |
|  |  | Men | " | " | 5.86 | < .001* | 23 | [0.16, 0.33] | 1.20 | 1.24 ± 0.20 | 1.00 ± 0.00 |
|  | Doxycycline | Women | " | " | 4.84 | < .001* | 23 | [0.29, 0.99] | 0.99 | 1.20 ± 0.21 | 1.00 ± 0.00 |
|  |  | Men | " | " | 7.51 | < .001* | 23 | [0.24, 0.42] | 1.53 | 1.33 ± 0.21 | 1.00 ± 0.00 |
