# Supplementary material for: Effect of the Matrix Metalloproteinase Inhibitor Doxycycline on Human Trace Fear Memory
Source: eNeuro. 2023 Feb 23;10(2):ENEURO.0243-22.2023. doi: 10.1523/ENEURO.0243-22.2023 (PMC9961363; doi:10.1523/ENEURO.0243-22.2023)
Supplement: Extended Data Figure 3-4 — Acquisition independent t test between CS+/CS− difference for placebo and doxycycline group per gender, not corrected for multiple comparisons. P = Placebo, D = Doxycycline Download Figure 3-4, DOC file. [file enu-eN-NRS-0243-22-s05.doc]

| **Figure 3-4** | |  |  |  |  |  |  |  |  |  |  | |
| --- | --- | --- | --- | --- | --- | --- | --- | --- | --- | --- | --- | --- |
| Acquisition independent t-test between CS+/CS- difference for placebo and doxycycline group per gender, not corrected for multiple comparisons | | | | | | | | |  |  |  | |
| P = Placebo, D = Doxycycline | |  |  |  |  |  |  |  |  |  |  | |
|  |  |  |  |  |  |  |  |  |  |  |  | |
|  |  |  |  |  |  |  |  |  |  | **Mean CSplus - CSminus (± SD)** | | |
| **Measure** | **Group** | **Gender** | **Specification** | **averaged** | **t-statistic** | **p** | **df** | **95% CI** | **cohen's d** | **Placebo** | | **Doxycycline** |
| SCR | P vs. D | Women | to CS presentation | trial 1-20 | 0.93 | 0.36 | 41.40 | [-0.09, 0.25] | 0.27 | 0.22 ± 0.33 | | 0.14 ± 0.24 |
|  |  |  | during trace interval | " | -1.37 | 0.18 | 45.31 | [-0.61, 0.12] | 0.40 | 0.35 ± 0.59 | | 0.60 ± 0.66 |
|  |  |  | to US presentation | " | 0.15 | 0.88 | 41.62 | [-0.45, 0.52] | 0.04 | 0.66 ± 0.96 | | 0.62 ± 0.69 |
|  | P vs. D | Men | to CS presentation | " | -1.75 | 0.088 | 41.99 | [-0.25, 0.02] | 0.50 | 0.12 ± 0.19 | | 0.24 ± 0.26 |
|  |  |  | during trace interval | " | -0.88 | 0.38 | 45.10 | [-0.54, 0.21] | 0.25 | 0.53 ± 0.69 | | 0.70 ± 0.60 |
|  |  |  | to US presentation | " | 0.73 | 0.47 | 43.57 | [-0.31, 0.65] | 0.21 | 0.64 ± 0.72 | | 0.46 ± 0.92 |
| PSR | P vs. D | Women | fitted | trial 1-20 | 1.05 | 0.30 | 43.97 | [-0.06, 0.20] | 0.30 | 0.27 ± 0.26 | | 0.20 ± 0.21 |
|  | P vs. D | Men | " | " | -1.40 | 0.17 | 45.89 | [-0.21, 0.04] | 0.41 | 0.24 ± 0.20 | | 0.33 ± 0.21 |
