# Supplementary material for: Effect of the Matrix Metalloproteinase Inhibitor Doxycycline on Human Trace Fear Memory
Source: eNeuro. 2023 Feb 23;10(2):ENEURO.0243-22.2023. doi: 10.1523/ENEURO.0243-22.2023 (PMC9961363; doi:10.1523/ENEURO.0243-22.2023)
Supplement: Extended Data Figure 5-1 — Mediation analysis for effect of sex on doxycycline serum levels mediated by weight. Download Figure 5-1, DOC file. [file enu-eN-NRS-0243-22-s13.doc]

| **Figure 5-1** |  |  |  |  |
| --- | --- | --- | --- | --- |
| Mediation Analysis for effect of sex on doxycycline serum levels mediated by weight | | | | |
|  | **Estimate** | **95% CI** | | **p-value** |
| ACME | 0.31 | [-0.03, 0.72] | | 0.078 |
| ADE | 0.82 | [0.10, 1.55] | | 0.028 |
| Total Effect | 1.13 | [0.57, 1.71] | | 0.004 |
| Prop. Mediated | 0.28 | [-0.04, 0.83] | | 0.082 |
